# New Weighting Methods for Phylogenetic Tree Reconstruction Using Multiple Loci

**DOI:** 10.1007/s00239-012-9513-4

**Published:** 2012-08-08

**Authors:** Kazuharu Misawa, Fumio Tajima

**Affiliations:** 1 Research Program for Computational Science, Research and Development Group for Next-generation Integrated Living Matter Simulation, Fusion of Data and Analysis Research and Development Team, RIKEN, 1-7-22 Suehiro-cho, Tsurumi, Yokohama, 230-0045 Japan; 2 Department of Biological Sciences, Graduate School of Science, The University of Tokyo, Tokyo, Japan

**Keywords:** Phylogeny reconstruction, Weighting methods, Computer simulation

## Abstract

Efficient determination of evolutionary distances is important for the correct reconstruction of phylogenetic trees. The performance of the pooled distance required for reconstructing a phylogenetic tree can be improved by applying large weights to appropriate distances for reconstructing phylogenetic trees and small weights to inappropriate distances. We developed two weighting methods, the modified Tajima–Takezaki method and the modified least-squares method, for reconstructing phylogenetic trees from multiple loci. By computer simulations, we found that both of the new methods were more efficient in reconstructing correct topologies than the no-weight method. Hence, we reconstructed hominoid phylogenetic trees from mitochondrial DNA using our new methods, and found that the levels of bootstrap support were significantly increased by the modified Tajima–Takezaki and by the modified least-squares method.

**Electronic supplementary material:**

The online version of this article (doi:10.1007/s00239-012-9513-4) contains supplementary material, which is available to authorized users.

## Introduction

A phylogenetic tree is a powerful tool for investigating the evolutionary history of organisms and genes. Nowadays, molecular phylogenetic analysis has become one of the most important methods for not only comparative studies of organisms (Harvey and Pagel 1991) but also for the study of the evolution of genes (Nei et al. 2008). Molecular phylogenetic trees can be used for clustering gene families (Misawa and Tajima 2000). Molecular phylogenetic analysis has gained importance because of advances in DNA sequencing techniques and sequence databases.

At present, a large number of DNA and amino acid sequences are available for molecular phylogenetic studies (Kuma and Miyata 1994; Misawa and Janke 2003; Murphy et al. 2001; Nozaki et al. 2009). These sequences may have different amounts of information about the phylogenetic relationships of the organisms in the study, and different amounts of noise obscuring those relationships (Russo et al. 1996). Phylogenetic information is encoded in the DNA or protein sequences of contemporary species in a manner that allows the information from data such as DNA sequences to be used to trace the history back to the most recent common ancestor of the species (Liu et al. 2009).

The method of phylogenetic inference currently used in molecular phylogenetics can be classified into four major groups: distance methods, maximum likelihood methods, Bayesian methods, and parsimony (Nei and Kumar 2000). In distance methods, an evolutionary distance is computed for all pairs of sequences, and a phylogenetic tree is constructed from pairwise distances such as neighbour joining (NJ) method (Saitou and Nei 1987). When the phylogenetic tree is reconstructed using the distance methods, the error in phylogenetic tree reconstruction can be reduced by applying large weights to distances with large information and small noise and small weights to noisy distances with small information (Bull et al. 1993). To recover correct phylogenies, many authors have developed methods to determine the weights for transitional and transversional substitutions in cases where the Kimura 2-parameter model is used. Tajima and Takezaki (1994) defined an accuracy index for evolutionary distance and determined the weights that maximize the accuracy. Goldstein and Pollock (1994) used a least-squares method to determine the weights that produce a minimum-variance estimator from transition and transversion substitutions. Unfortunately, there is no consensus on the method for pooling distance data obtained for multiple loci (Dutilh et al. 2004; Huelsenbeck et al. 1996).

The purpose of this study is to develop improved methods to weight distances from different genes for accurate reconstruction of phylogenetic trees. We have modified the Tajima–Takezaki method and the Goldstein and Pollock method for multiple genes. Two new methods developed were a modified Tajima–Takezaki method and a modified least-squares method. We used computer simulations to compare these two new methods to the least-squares method and a no-weight method, evaluating their abilities to recover the correct tree topology. In this study, “efficiency” means the ability to recover the correct tree topology. We determined the weights required to pool the distances estimated for the mitochondrial genes and reconstructed a hominoid phylogenetic tree.

## Materials and Methods

### Weighting Methods

In this paper, we used four weighting methods: the no-weight method, the least-squares method, the modified Tajima–Takezaki method, and the modified least- squares method. In the least-squares method, each locus was weighted by the average reciprocal of the sampling variances for the estimates of evolutionary distances for that locus (Lynch 1999). Goldstein and Pollock (1994) also followed this approach to obtain an efficient distance by pooling transitional and transversional distances to recover correct phylogenetic trees from DNA sequences. The purpose of Goldstein and Pollock’s method (1994) is to bring transversional distance and transitional distance together. Our purpose is to bring together distances from several loci. The purpose of Lynch (1999) is to obtain the divergence time, while our purpose is to reconstruct phylogenetic trees. Therefore, the least-squares method used by us differs from those reported by Goldstein and Pollock (1994) and Lynch (1999). The modified Tajima–Takezaki method maximizes the accuracy index (Tajima and Takezaki 1994) of the pooled distance, whereas the least-squares methods minimizes its variance (Goldstein and Pollock 1994). The modified least-square method is similar to the least-square method, but it puts a single weight for all OTU pairs for one gene, insuring a suboptimal weight will be used with all but one of OTU pairs, while the least-square method puts one weight for each OTU pair for one gene. The details of these weighting methods were described in Supplemental Materials.

### Computer Simulation

Computer simulations were conducted to compare the efficiencies of weighting methods for phylogeny reconstruction. Since the efficiencies of the weighting methods would depend on the tree topology and branch lengths (Goldstein and Pollock 1994; Tajima and Takezaki 1994; Pollock and Goldstein 1995), the simulations were performed under various conditions (Supplemental Materials).

**Fig. 1:**
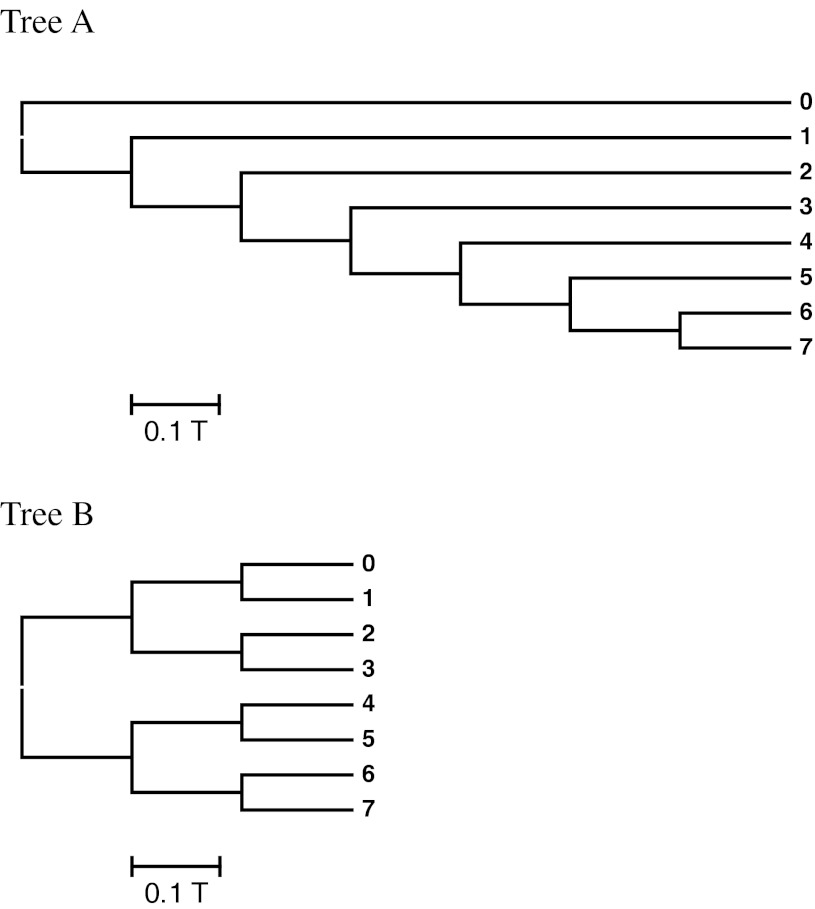
The model trees. **Tree A** is an asymmetric tree and **Tree B** is a symmetric tree. T is the unit of time. When the branch length is 0.1 T, the expected number of substitutions per site is 0.1 T*u*

We used 2 model trees as shown in Fig. 1. T is the time unit in the simulation. Tree A is an asymmetric tree and tree B is a symmetric tree. These trees are basically the same as those used by Tateno, Nei, and Tajima (1982).

Let us define *u*(*h*) as the substitution rate per *T* per site of gene *h*. The *u*(*h*) value was assumed to be the same for all sites of gene *h*. In order to introduce heterogeneity in the evolutionary rate among genes, *u*(*h*) was assumed to follow the gamma distribution (Yang 1996), where *a* and *b* are the parameters that determine the shape of the gamma distribution. Let us denote the expected value of *u*(*h*) as *u*. The expectation and variance of *u*(*h*) are given by *E*[*u*(*h*)] = *u* = $\frac{a}{b}$, and *V*[*u*(*h*)] = $\frac{{u^{2} }}{a}$, respectively. Note that when the value of *a* is infinity, there is no rate variation among the loci. Gamma-distributed random numbers were generated using the algorithm described by Ahrens and Dieter (1974).

In the computer simulation, sequences of 10 loci were generated (see Supplemental Materials). We conducted 2 sets of computer simulations. One is to examine the effects of the rate variation and the weighting methods on phylogenetic tree reconstruction, and the other is to examine the effects of the average rate and the weighting methods on phylogenetic tree reconstruction. When the former was investigated by computer simulation, *u* was fixed to 0.5, and *a* was incremented by 0.1 from 0.1 to 1.9. When the latter was investigated by computer simulation, *u* was incremented by 0.1 from 0.1 to 1.9, and *a* was fixed to 0.5.

To estimate the number of substitutions per site, Poisson distance was used for protein sequences, and Kimura’s (1980) 2-parameter distance was used for tRNA sequences. To determine the pooled distances, four sets of methods, namely, the no-weight method, least-squares method modified Tajima–Takezaki method, and modified least-squares method, were used. Gene names and gene lengths are shown in Supplementary Materials. For each gene, the weights obtained using the modified Tajima–Takezaki method and the modified least-squares method are shown in Supplementary Materials.

By the generated amino acid sequences, we obtained the evolutionary distances among OTUs using the Poisson distance (see Supplemental Material). By the generated DNA sequences, we obtained the evolutionary distances among OTUs using the Kimura’s (1980) 2 parameter distance. All the distances obtained using the methods described above were pooled. We used the following 4 pooling methods: the no-weight method, least-squares method, modified Tajima–Takezaki method, and modified least-squares method.

Finally, the efficiencies of weighting methods were compared. The trial simulation was repeated 10,000 times for each set of parameters, and the proportion of trials that yielded the correct tree topology (PC) was obtained. We also compared the topological distances (Rzhetsky and Nei 1992) between the correct tree and the reconstructed tree (dT). See Supplemental Material.

### Application in Hominoid Mitochondrial Phylogeny

We compared these methods in construction of a hominoid phylogenetic tree to the no-weight and least-squares methods using mitochondrial genes. Since the mitochondrial phylogeny in hominoid is well established (Horai et al. 1995), we reconstruct hominoid phylogeny using mitochondrial genes to compare the methods described above. We used 13 protein sequences as well as 22 tRNA sequences of mitochondrial DNA (mtDNA) of four hominoid species, namely, orangutan (*Pongo pygmaeus abelii*), gorilla (*Gorilla gorilla*), bonobo (*Pan paniscus*), and human (*Homo sapiens*). The accession numbers for the mitochondrial DNA sequences of orangutan, gorilla, bonobo, and human are X97707, D38114, D38116, and D38112, respectively. Mitochondrial sequences were aligned using the MAFFT program (Katoh et al. 2002). The complete deletion option (Nei and Kumar 2000) was used for the gapped sites in the reconstructed phylogenetic trees. Alignments are available at http://sourceforge.jp/projects/parallelgwas/releases/?package_id=9706.

To estimate the number of substitutions per site, in Supplementary Text (21) was used for protein sequences, and Eq. (26) in Supplementary Text was used for DNA sequences. To determine the pooled distances, four sets of methods, namely, the no-weight method, least-squares method, modified Tajima–Takezaki method, and modified least-squares method, were used. Gene names and gene lengths are shown in Supplementary Table 2. The NJ trees were reconstructed using the pooled distances. We performed the bootstrap test for phylogenetic relationships (Felsenstein 1985). Bootstrap resampling was performed 10,000 times.

## Results

### Computer Simulation

**Fig. 2:**
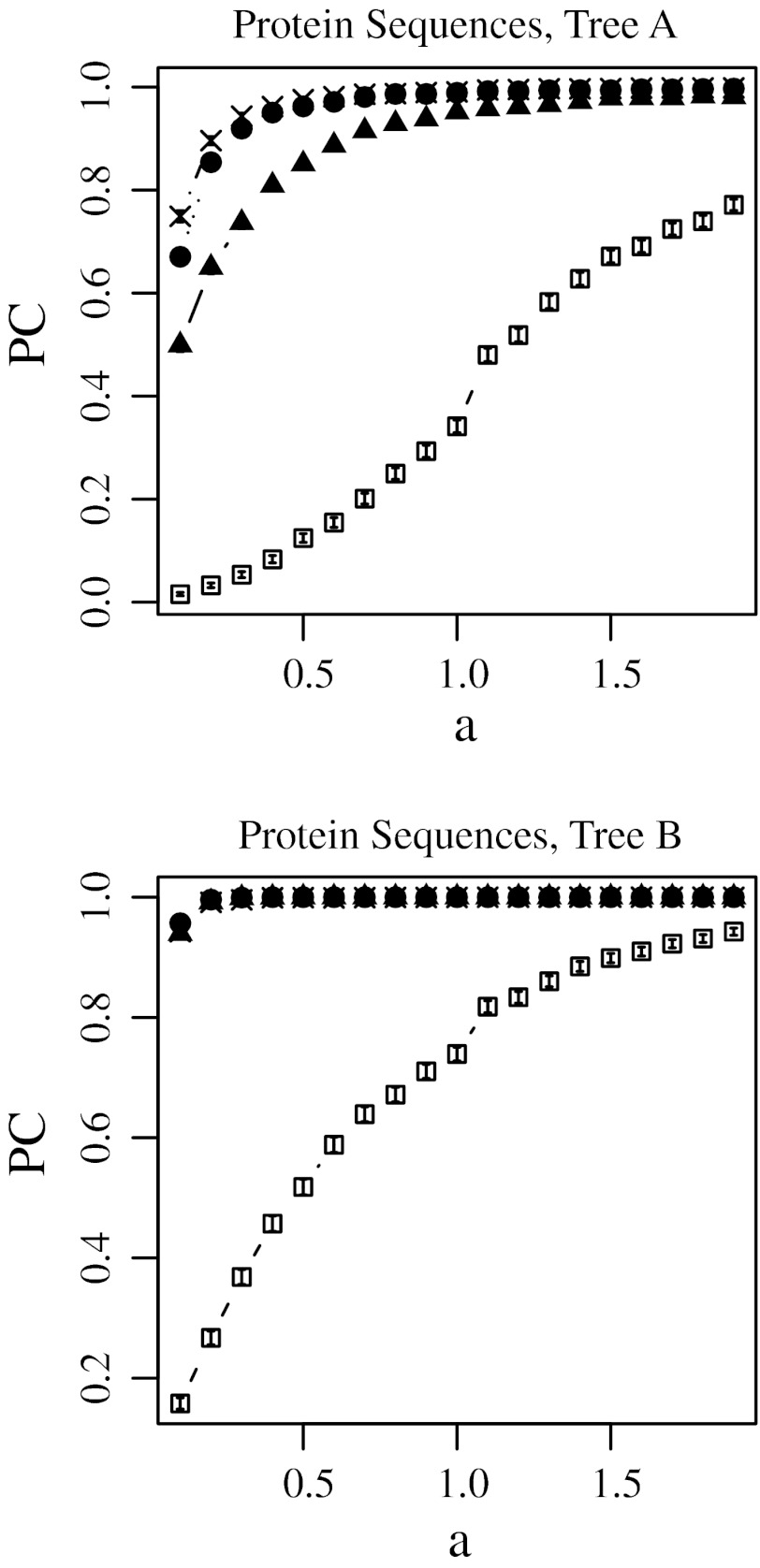
Proportion of trials yielding the correct tree topology (PC) using the no-weight method (*filled triangle*), the least-squares method (*open square*), the modified Tajima–Takezaki method (*filled circle*), and the modified least-squares method (*cross*) when protein sequences were simulated and the Poisson distances are used. In this figure, *u* was fixed to 0.5 and *a* was incremented, where *u* is the average mutation rate and *a* is the gamma-shape parameter. 99 % confidence intervals are also shown

**Fig. 3:**
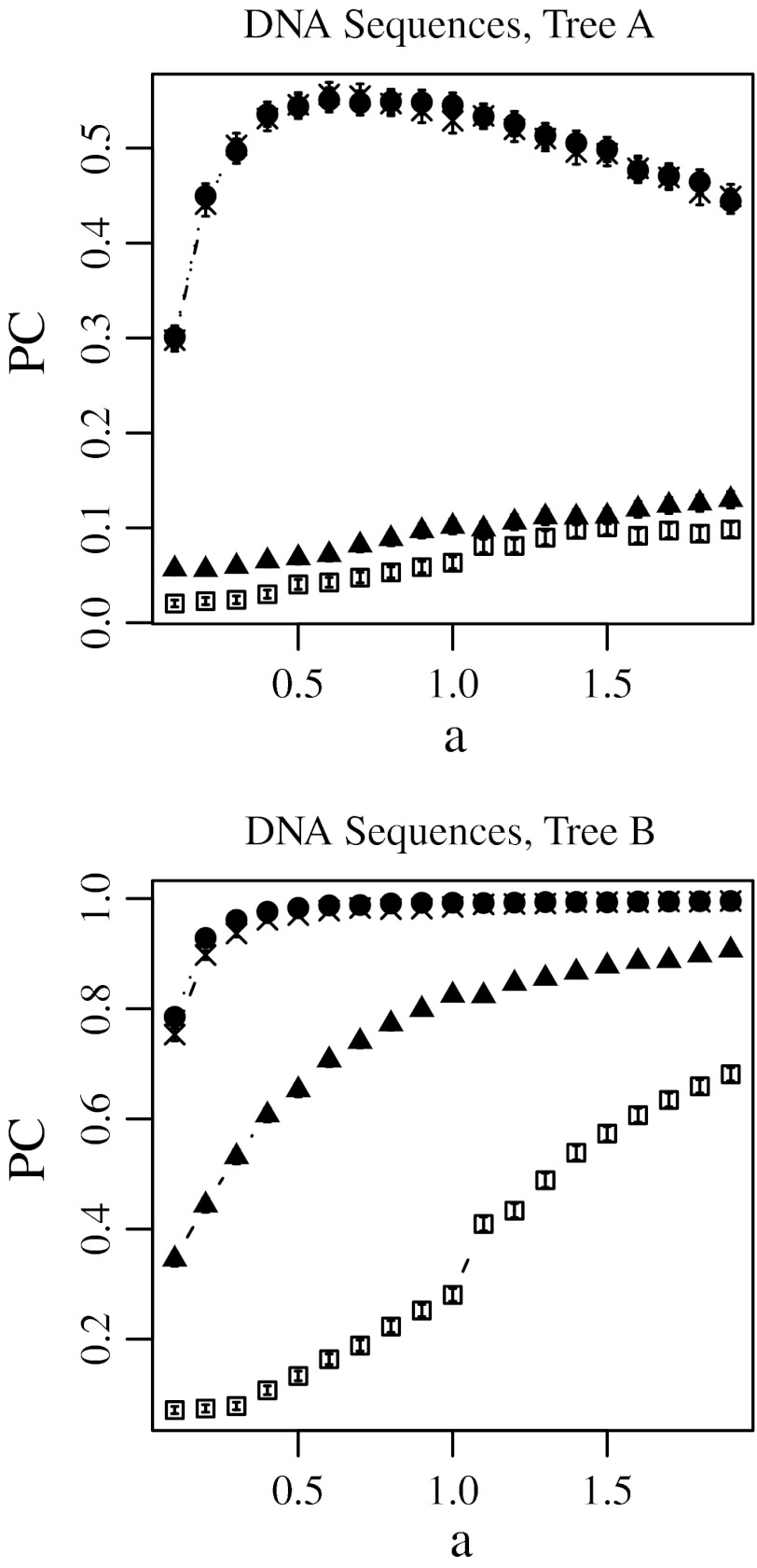
Proportion of trials yielding the correct tree topology (PC) using the no-weight method (*filled triangle*), the least open square method (*open square*), the modified Tajima–Takezaki method (*filled circle*), and the modified least-squares method (*cross*) when DNA sequences were simulated and the Kimura’s (1980) 2-parameter distances are used. In this figure, *u* was fixed to 0.5 and *a* was incremented, where *u* is the average mutation rate and *a* is the gamma-shape parameter. 99 % confidence intervals are also shown

**Fig. 4:**
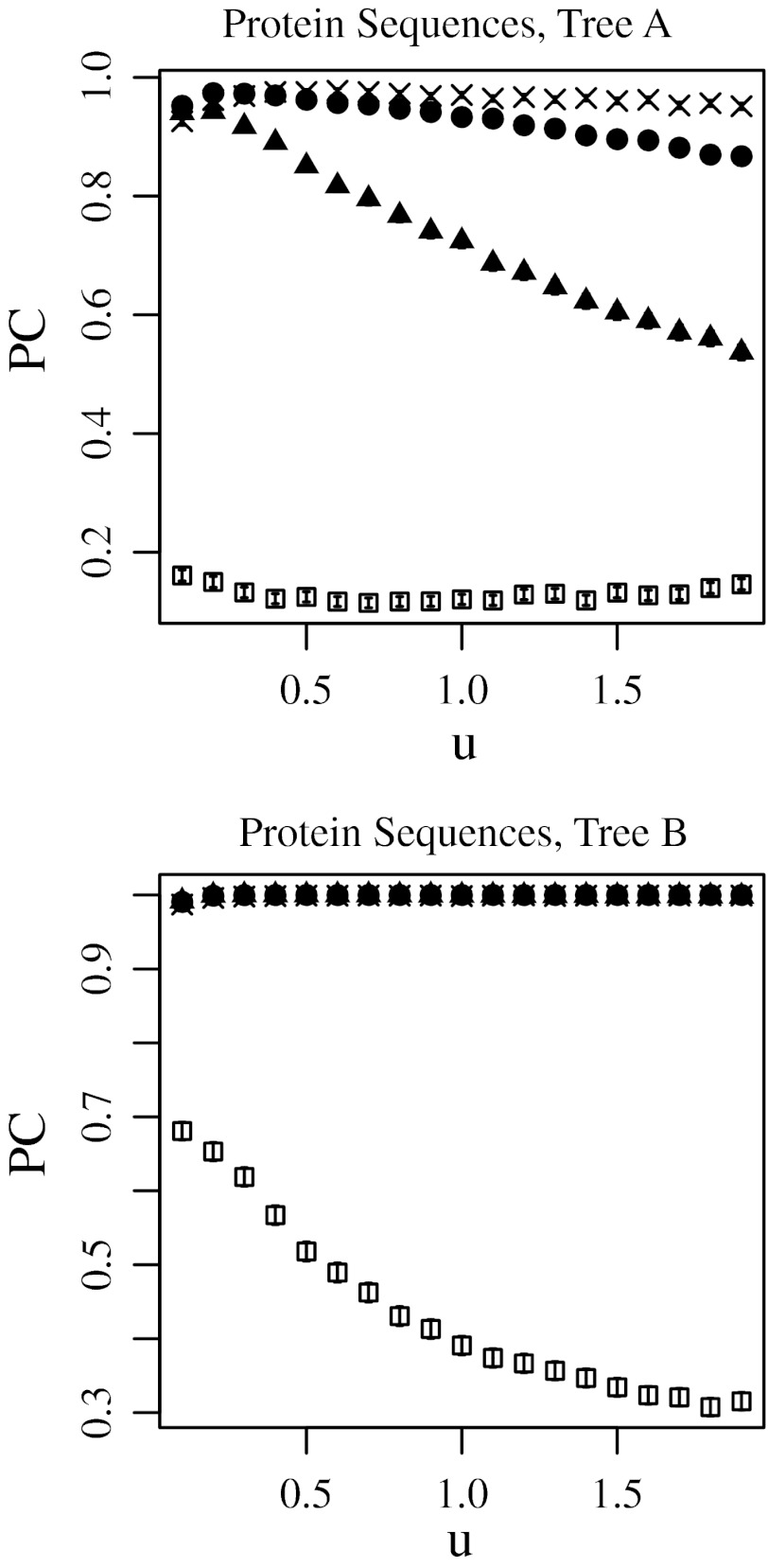
Proportion of trials yielding the correct tree topology (PC) using the no-weight method (*filled triangle*), the least open square method (*open square*), *t* modified Tajima–Takezaki method (*filled circle*), and the modified least-squares method (*cross*) when protein sequences were simulated and the Poisson distances are used. In this figure, *u* was incremented and *a* was fixed to 0.5, where *u* is the average mutation rate and *a* is the gamma-shape parameter. 99 % confidence intervals are also shown

**Fig. 5:**
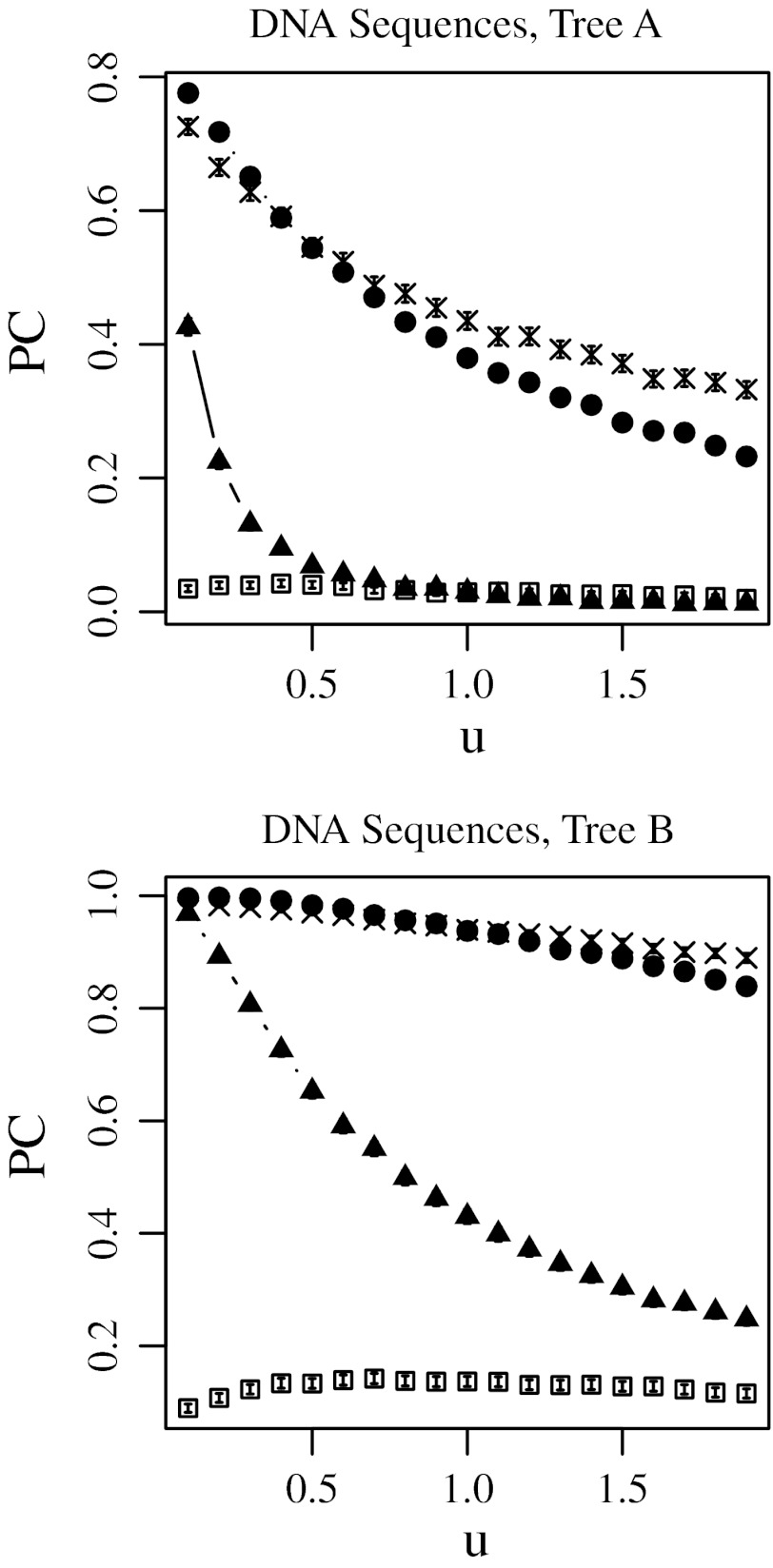
Proportion of trials yielding the correct tree topology (PC) using the no-weight method (*filled triangle*), the least open square method (*open square*), modified Tajima–Takezaki method (*filled circle*), and the modified least-squares method (*cross*) when DNA sequences were simulated and the Kimura’s (1980) 2-parameter distances are used. In this figure, *u* was incremented and *a* was fixed to 0.5, where *u* is the average mutation rate and *a* is the gamma-shape parameter. 99 % confidence intervals are also shown

Figures 2, 3, 4, and 5 show the proportions of trials in which correct topology reconstructions were obtained using the no-weight method, least-squares method, modified Tajima–Takezaki method, and modified least-squares method. Supplementary Fig. S1–S4 show the mean of the topological distances between the model trees and reconstructed trees under the same condition. We observed a strong correlation between the results obtained by PC and those obtained by dT. Because PC and dT give such similar results, we chose to present PC in the results section. Figures 2 and 4 show the results obtained when protein sequences were simulated, whereas Figs. 3 and 5 show the results obtained when DNA sequences were simulated.

In Figs. 2 and 3, the substitution rate *u* was fixed to 0.5, and the gamma-shape parameter *a* was incremented. In Figs. 4 and 5, *u* was incremented, and *a* was fixed to 0.5. Note that *a* is inversely proportional to the variation in substitution rates among loci as defined in Eqs. (11) and (12) in Supplementary Text.

### The No-Weight Method

The filled triangles in Figs. 2, 3, 4, and 5 indicate the cases in which the correct topology was reconstructed by the no-weight method. Figures 2 and 3 show that for the no-weight method, the proportion of trials yielding the correct phylogenetic tree increased as the rate variation decreased. Figures 4 and 5 show that for the no-weight method, the proportion of trials yielding the correct phylogenetic tree decreased as the substitution rate increased. These figures show that it was more difficult to reconstruct tree A than it was to reconstruct tree B (see “Discussion” section).

### The Least-Squares Method

The open squares in Figs. 2, 3, 4, and 5 indicate the cases in which the correct topology was reconstructed by the least-squares method. These results suggest that the no-weight method performs better than the least-squares method. Figures 2 and 3 show that when the least-squares method was used, the proportion of cases in which the correct phylogenetic tree was recovered increased as the rate variation decreased. In Figs. 4 and 5, we can see that the least-squares method performs poorly when *a* was fixed at 0.5, except in the case of tree B, which was constructed using protein sequences and at a small substitution rate.

### Modified Tajima–Takezaki Method and Modified Least-Squares Method

The filled circles in Figs. 2, 3, 4, and 5 indicate the cases in which the correct topology was reconstructed using the modified Tajima–Takezaki method. We can see that for the reconstruction of phylogenetic trees, the modified Tajima–Takezaki method is better than the no-weight and least-squares methods. Moreover, these figures show that the modified Tajima–Takezaki method is better than the no-weight method for the reconstruction of phylogenetic trees, especially when the extent of rate variation among loci is large.

The crosses in Figs. 2, 3, 4, and 5 indicate the cases in which the correct topology was reconstructed using the modified least-squares method. We can see that for the reconstruction of phylogenetic trees, the modified least-squares method is better than the no-weight method and the least-squares method, and is as good as the modified Tajima–Takezaki method.

In Fig. 3, we can see that the proportion of cases wherein tree A was recovered using DNA sequences was the highest when *a* was approximately 0.6, in case of both the modified Tajima–Takezaki method and the modified least-squares method. Such a peak was not observed when the no-weight or the least-squares method was used. These peaks indicate that the modified Tajima–Takezaki and the modified least-squares methods appropriately pool distances from both slow- and fast-evolving loci.

In Figs. 4 and 5, we can see that when the modified Tajima–Takezaki or the modified least-squares method was used, the proportion of cases in which the correct phylogenetic tree was recovered decreased as the substitution rate increased. However, the rate of decrease as a function of the substitution rate was smaller in the case of these 2 methods than in the case of the no-weight method.

In cases when *k* is large, the modified least-squares method is better than the modified Tajima–Takezaki method. Conversely, in cases where the divergence of sequences is small, the modified Tajima–Takezaki method is better than the modified least-squares method.

### Application in Hominoid Mitochondrial Phylogeny

**Fig. 6:**
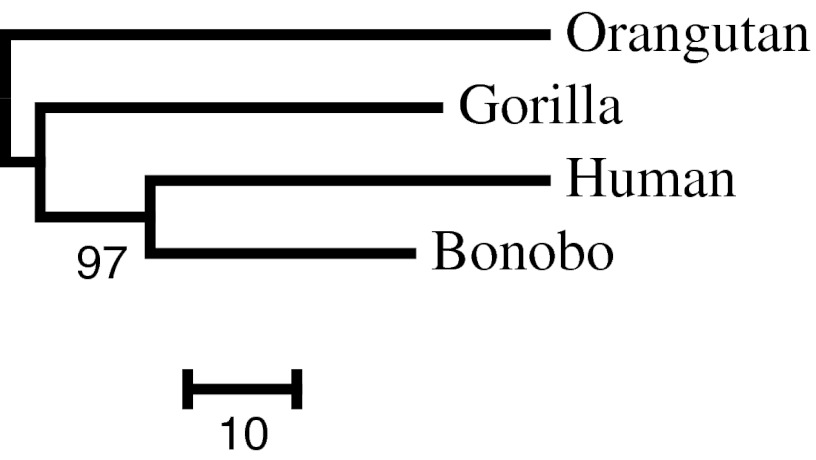
NJ tree among hominoids. Distances were obtained using Poisson distance from mitochondrial protein sequences and pooled using the modified Tajima–Takezaki method. The no-weight method, the least-squares method, and the modified least-squares method yielded the same tree topology. Distances obtained using Kimura 2-parameter method from mitochondrial tRNA sequences and pooled by the no-weight method, by the least-squares method, by the modified Tajima–Takezaki method, and by the modified least-squares method also yielded the same tree topology. The *number* below the branch leading to the human^bonobo cluster is the bootstrap support value (%) when mitochondrial protein sequences and the no-weight method were used

The no-weight method uses gene lengths as defined in Eq. (1) in Supplementary Text; the gene lengths are shown in Supplementary Table 2. The NJ tree reconstructed using the no-weight method and mitochondrial protein sequences is shown in Fig. 6. The topology obtained using the no-weight method and all the other weighting methods was the same as that obtained in a previous study (Horai et al. 1995), regardless of whether mitochondrial protein sequences or mitochondrial tRNA sequences were used. Henceforth, the clusters will be referred to by the names of two species involved; for instance, in the case of the OTUs *i* and *j*, the *i*^*j* cluster corresponds to the cluster of all descendants of the common ancestors of *i* and *j*. The no-weight, least-square, modified Tajima–Takezaki, and modified least-squares methods were used to determine the number of trials in which the human^bonobo cluster was recovered among 10,000 bootstrap resampling trials when mitochondrial protein sequences or mitochondrial tRNA sequences were used (Table 1). For each gene, the weights obtained using Eq. (5) in Supplementary Text for the modified Tajima–Takezaki method and Eq. (10) in Supplementary Text for the modified least-squares method are shown in Supplementary Table 2.

**Table 1:** Bootstrap support values of the human^bonobo cluster

Weighting methods	Proteins	tRNAs
No-weight method	9,725	9,589
Least-squares method	9,808*	6,174^†^
Modified Tajima–Takezaki’s Method	9,833*	9,758*
Modified least-squares method	9,995*	9,372

* Significantly larger (Fisher exact test, *P* < 0.01) than the bootstrap support value of the no-weight method

^†^Significantly smaller (Fisher exact test, *P* < 0.01) than the bootstrap support value of the no-weight method

The number of trials in which the human^bonobo cluster was recovered using the least-squares method and mitochondrial protein sequences was significantly higher than that recovered using the no-weight method (Fisher’s exact test, *P* < 0.01). The number of trials in which the human^bonobo cluster was recovered using the least-squares method and mitochondrial tRNA sequences was significantly lower than that recovered using the no-weight method (Fisher’s exact test, *P* < 0.01).

The number of trials in which the human^bonobo cluster was recovered using the modified Tajima–Takezaki method and mitochondrial protein sequences was significantly higher than those recovered using the no-weight methods (Fisher’s exact test, *P* < 0.01). The number of trials in which the human^bonobo cluster was recovered using the modified Tajima–Takezaki method and mitochondrial tRNA sequences was also significantly higher than that recovered using the no-weight method (Fisher’s exact test, *P* < 0.01).

The number of trials in which the human^bonobo cluster was recovered using the modified least-squares method and mitochondrial protein sequences was significantly higher than that recovered using the no-weight method (Fisher’s exact test, *P* < 0.01). The number of trials in which the human^bonobo cluster was recovered using the modified least-squares method and mitochondrial tRNA sequences was smaller than when the no-weight method was used; however, these values did not significantly differ.

## Discussion

We developed two weighting methods, the modified Tajima–Takezaki method and the modified least-squares method, for reconstructing phylogenetic trees for multiple loci. Computer simulations showed that the new methods are more efficient than the no-weight method and the least-squares methods for reconstructing phylogenetic trees. We compared these methods in construction of a hominoid phylogenetic tree to the no-weight and least-squares methods using mitochondrial genes.

### No-Weight Method

Computer simulation showed that when the no-weight method was used, the correct tree was obtained more frequently as the rate variation decreased. When OTUs are diverged, the results obtained using Kimura’s (1980) 2-parameter distance are worse than those obtained using other distances such as Jukes and Cantor’s (1969) distance (Goldstein and Pollock 1994; Tajima and Takezaki 1994). As mentioned above, Tajima and Takezaki (1994) and Goldstein and Pollock (1994) have independently developed distances that are more efficient than Kimura’s (1980) 2-parameter distance for the reconstruction of phylogenetic trees. Because we did not intend to compare the distance methods, we only used distances that could be measured easily, namely, the Poisson distance (Zuckerkandl and Pauling 1965) and Kimura’s 2-parameter distance (Kimura 1980). One of the authors (KM) previously conducted computer simulations, and found that Tajima and Takezaki’s (1994) distance, Goldstein and Pollock’s (1994) distance, and Jukes and Cantor’s (1969) distance are better than Kimura’s (1980) 2-parameter distance when distances from multiple loci are pooled (Misawa 2000). These results suggest that increasing the accuracy of the distance obtained from each gene increases the efficiency in reconstructing the phylogenetic tree of the pooled distance. The choice of the model is important for phylogenetic reconstruction, as has been previously pointed out (Sullivan and Joyce 2005).

### Least-Squares Method

Computer simulations showed that the least-squares method is worse than the no-weight method. This may be because the estimate of sampling variance is strongly correlated to the estimates of the number of substitutions obtained from DNA or protein sequences [see Eqs. (13–27) in Supplementary Text]. The estimates of the number of substitution are usually not the same for all genes because of the sampling variances. Weighting by the least-square method was less accurate at reconstructing phylogeny because it uses inaccurate variance correction. This may be the reason the least-squares method yields unsatisfactory results as compared to the no-weight method.

When the least-squares method was applied to the mitochondrial protein sequences, the bootstrap support value for the human^bonobo cluster was significantly larger than that obtained using the no-weight method (Table 1). For proteins, the average of the estimates of the number of amino acid substitutions per site in orangutans and humans is 0.12 and the variance is 0.0052. Using Eq. (12) in Supplementary Text, we can estimate *a* for mitochondrial proteins as 2.68. Thus, the situation is similar to the computer simulation wherein *u* was small and *a* was large, using protein sequences. As we can see from the results of computer simulations, the modified Tajima–Takezaki method and the modified least-square method work well when *u* was small and *a* was large (Figs. 2, 4).

When the least-squares method was applied to the tRNA sequences, the bootstrap support value for the human^bonobo cluster was significantly smaller than that obtained using the no-weight method (Table 1). The average of the estimates of the number of tRNA substitutions per site in orangutans and humans is 0.14 and the variance is 0.010. Using Eq. (12) in Supplementary Text, we can estimate *a* for mitochondrial tRNAs to be 1.92. This situation corresponds to the computer simulation wherein *u* was small and *a* was large, using DNA sequences. This is why the least-squares method gave a poor result.

### Modified Tajima–Takezaki Method

The results of computer simulations showed that the modified Tajima–Takezaki method gives better results than the no-weight method for reconstructing phylogenetic trees. By DNA sequences, the modified Tajima–Takezaki method yielded the correct tree most frequently when *a* was approximately 0.6 (Fig. 3), probably because the sequences were efficiently pooled and the noise from inappropriate genes was reduced in the modified Tajima–Takezaki method. This peak was also observed when the modified least-squares method was used, but not observed when the no-weight method and the least-squares method were used.

The modified Tajima–Takezaki method is based on the rate constancy (molecular clock) of all OTUs. The rate constancy was assumed for all OTUs in the model trees, A and B, used in the computer simulations. We also conducted computer simulations without assuming rate constancy. We found that both the modified Tajima–Takezaki method and the modified least-squares method yield the correct tree more often than the no-weight and least-squares methods, even when rate constancy is not assumed (Supplementary Figs. S5–S8). Thus, the modified Tajima–Takezaki method is applicable in cases with and without rate constancy.

When the modified Tajima–Takezaki method was applied to the mitochondrial protein sequences and tRNA sequences, the bootstrap support value for the human^bonobo cluster was significantly larger than that obtained using the no-weight method. This result is consistent with the results of the computer simulations.

### Modified Least-Squares Method

Computer simulations showed that the modified least-squares method is always better than the no-weight method and as good as the modified Tajima–Takezaki method for reconstructing phylogenetic trees. The original least-squares method is much worse than the modified least-squares method. When all genes have the same expected values, weighting using Eq. (2) in Supplementary Text yields distances with the same expected value, close to minimum variance. However, when all the values are not same, the expected value obtained using Eq. (2) in Supplementary Text differs from the average value. Therefore, the least-squares method yields unsatisfactory results as compared to the no-weight method. On the contrary, the modified least-squares method provides a single weight for each gene. These results suggest that allotting a single weight for each gene by the modified least-squares method is better than allotting different weights for the OTUs of all genes by the original least-squares method, especially when the rate variation among loci is large.

Computer simulations also showed that in the case of highly divergent sequences, the modified least-squares method is better than the modified Tajima–Takezaki method. Conversely, in cases where the divergence of sequences is small, the modified Tajima–Takezaki method is slightly better than the modified least-squares method. This relationship is similar to the relationship between the Tajima and Takezaki (1994) method and the Goldstein and Pollock (1994) method for DNA sequences (Pollock and Goldstein 1995). In other words, the modified least-squares method may be too sensitive for distances close to 0. Among the tRNA sequences used in this study, the sequence of tRNA-Met in humans is exactly the same as that in bonobos and orangutans. In gorillas, this sequence is different from that in the other 3 species. In such a case, not only *k*(*h*, *i*, *j*) but also *V*[*k*(*h*, *i*, *j*)] is close to 0, and Eq. (10) in Supplementary Text gives large weights to tRNA-Met (see Supplementary Table 2). This sensitivity may have caused the decrease in the bootstrap support value of the human^bonobo cluster when the modified least-squares method was applied to the mitochondrial tRNA sequences. The nucleotide differences of tRNA-Ala, tRNA-Leu(CUN), tRNA-Gln, and tRNA-Leu(UUR) between human and bonobo are one, so that the weights that put on these tRNAs were also high (see Supplementary Table 2). When we removed tRNA-Met, tRNA-Ala, tRNA-Leu(CUN), tRNA-Gln, and tRNA-Leu(UUR), the number of trials in which the human^bonobo cluster was recovered using the modified least-squares method and mitochondrial tRNA sequences was 9,640. This number was larger than that when we used all tRNAs as shown in Table 1. When we removed tRNAs whose nucleotide differences between human and bonobo are larger than one, the number of trials in which the human^bonobo cluster was recovered using the modified least-squares method got smaller (data not shown). These results also suggest that the generalized least-squares approach accounting for differences among genes may be slightly too sensitive for differences close to 0.

The average of the estimates of the number of amino acid substitutions per site in orangutans and humans is 0.12 and that of tRNA substitutions per site in orangutans and humans is 0.14. The estimate of *a* for mitochondrial proteins as 2.68 and that for mitochondrial tRNAs as 1.92. Thus, we conducted computer simulation corresponds to the situation in mitochondrial sequence. Since the longest distance among the OTU paris of tree A is 1.4 T and that of tree B is 0.6T, we fixed *u* to 0.1 and *a* was incremented by 0.1 from 0.1 to 2.9 (Supplementary Figs. S9–S12). The results were essentially the same as Figs. 2, 3, 4, and 5.

### Future Directions

In the simulation, we assume that the tree topology for all genes is identical, as shown in Fig. 1. We used mitochondrial genes that reflect the shared maternal history of organisms. We should note that gene trees differ from the species tree, because of ancestral polymorphisms, horizontal gene transfer, or gene duplications (Nakhleh et al. 2009). The effect of variations in the gene tree must be considered during the future studies on weighting.

In this paper, we used simple models such as the Jukes and Cantor (1969) model with 20 character states for amino acid substitutions. However, it would be more appropriate to generate protein sequences using more realistic models such as Dayhoff (Dayhoff et al. 1978), JTT (Jones et al. 1992), BLOSUM (Henikoff and Henikoff 1992), or Misawa and Kikuno (Misawa and Kikuno 2009). Further investigation is necessary to identify the best model.

The rate variation among the sites within each locus was not taken into consideration in the Poisson distance (Zuckerkandl and Pauling 1965) and the Kimura 2-parameter distance (Kimura 1980). However, the rate variation among sites is also important for phylogenetic reconstruction (Sullivan et al. 1995; Yang 1996). With regard to the program, it should be noted that the estimate of the rate variation among sites is also subject to sampling errors (Sullivan et al. 1999; Takezaki and Gojobori 1999). These sampling errors would affect the estimation of the distances and their variance. Because our weighting methods depend on the variation in evolutionary distances, sampling errors of rate variation must be taken into account. Pollock (1998) had developed an estimator of evolutionary distance with increased accuracy. His method deals explicitly with site-to-site rate variation, regions with biased nucleotide frequencies, and synonymous sites in protein-coding regions. This study also includes a methodology to obtain accurate distance estimates for large numbers of sequence regions evolving in different manners. In future, these features must be included into our method.

Recent studies have suggested that amino acid changes are affected by CpG hypermutability, so that amino acid substitutions as well as nucleotide substitutions are depending on the adjacent sites (Misawa et al. 2008). About 14 % of synonymous and nonsynonymous substitutions on human genes were caused by CpG hypermutability (Misawa and Kikuno 2009). It is still unclear how to estimate the sampling variance of evolutionary distance when the substitutions are depending on the adjacent sites and not time-reversible. Further study on substitution rates of DNA sequences and protein sequences is necessary.

## Conclusion

We developed two weighting methods, the modified Tajima–Takezaki method and the modified least-squares method, for reconstructing phylogenetic trees from multiple loci. The new methods are more suited to pool distances than the no-weight method and the least-squares method. The program for constructing a phylogenetic tree using these weighting methods is available at https://sourceforge.jp/projects/parallelgwas/releases/.

## Electronic supplementary material

Below is the link to the electronic supplementary material.

Supplementary material 1 (DOC 560 kb)

Supplementary material 2 (PDF 189 kb)

## Abbreviation

OTU: Operational taxonomy unit
