# Supplementary material for: New Weighting Methods for Phylogenetic Tree Reconstruction Using Multiple Loci
Source: J Mol Evol. 2012 Aug 8;75(1):1–10. doi: 10.1007/s00239-012-9513-4 (PMC3480593; doi:10.1007/s00239-012-9513-4)
Supplement: Supplementary file 1 — Supplementary material 1 (DOC 560 kb) [file 239_2012_9513_MOESM1_ESM.doc]

# Supplementary Materials

## **Weighting Methods**

In this section, we describe four weighting methods: the no-weight method, the least square method, and the modified Tajima-Takezaki method and the modified least square method. Supplementary Table 1 shows the list of variables and constants.

## The No-Weight Method

Let us denote the estimated number of substitutions per site of gene *h* between the operational taxonomic units (OTUs) *i* and *j* by $k_{t}(h,i,j)$at time *t*. When weighting methods are not used, the distance between the OTUs *i* and *j* can be obtained by the following equation:

$K(i,j)$ = $l(h)$ $k_{t}(h,i,j)$, (1)

where *l*(*h*) is the length of gene *h*. In other words, $K(i,j)$is the total number of substitutions between the OTUs *i* and *j*.

## The Least Square Method

In order to pool the data from all loci to obtain a single estimate of divergence, each locus was weighted by the average reciprocal of the sampling variances for the estimates of $k_{t}(h,i,j)$ for that locus (Lynch 1999). Goldstein and Pollock (1994) also followed this approach to get an efficient distance by pooling transitional and transversional distances to recover correct phylogenetic trees from DNA sequences. Goldstein and Pollock (1994) is to bring transversional distance and transitional distance together. Let us denote the variance of $k_{t}(h,i,j)$by $V[k_{t}(h,i,j)]$. By assigning 1/$V[k_{t}(h,i,j)]$ as the weight for the locus *h*, the pooled distance between the OTUs *i* and *j*, that is, $L(i,j)$, is obtained by the following equation:

$L(i,j)$ = $\frac{\sum_{h=1}^{N} w(h,i,j)k_{t}(h,i,j)}{\sum_{h=1}^{N} w(h,i,j)}$, (2)

where $w(h,i,j)=1/V[k_{t}(h,i,j)]$. Equation (2) is identical to equation (3) described by Lynch (1999). When all of $k(h,i,j)$ values are identical, weighting with the inverse of the sampling variances for the individual loci yields the distance that is expected to have close to minimum variance (see Discussion). It is worth noting that $w(h,i,j)$ is an increasing function of the gene length *l*(*h*) because $V[k_{t}(h,i,j)]$ is inversely proportional to *l*(*h*). It is also worth noting that $V[k_{t}(h,i,j)]$ is depending on $k_{t}(h,i,j)$ as described below.

## Modified Tajima-Takezaki Method

Let us assume OTUs *i* and *j* diverged at time *t*. Let us also assume OTUs *x* and *y* diverged at time *t* +$\Delta t$. The efficiency of evolutionary distances for reconstructing the correct tree topology depends on whether we can distinguish $k_{t}(h,i,j)$ from $k_{t+\Delta t}(h,x,y)$, for a given $\Delta t$(Tajima and Takezaki 1994), because reconstructing phylogenetic trees is determining the order of divergence. Note that $k_{t}(h,i,j)$ is a point estimator of the evolutionary distances at time *t*, and the width of confidence intervals of $k_{t}(h,i,j)$can be considered to be proportional to the standard deviation of $k_{t}(h,i,j)$. To distinguish $k_{t}(h,i,j)$ from $k_{t+\Delta t}(h,x,y)$, the latter must be outside the limits of the confidence interval of $k_{t}(h,i,j)$.

Tajima and Takezaki (1994) defined the accuracy index, $A[k_{t}(h,i,j)]$, as follows:

$A[k_{t}(h,i,j)]$ = $\frac{\frac{d}{dt}k_{t}(h,i,j)}{\sqrt{V[k_{t}(h,i,j)]}}$, (3)

where $\frac{d}{dt}k_{t}(h,i,j)$ is the time derivative of $k_{t}(h,i,j)$.

In this study, we propose a new weighting method to maximize the accuracy index. Let $M(i,j)$ be the pooled distance defined by the following equation:

$M(i,j)=\sum_{h=1}^{N} W(h)k_{t}(h,i,j)$, (4)

We found that *W*(*h*) that maximizes the accuracy index of $M(i,j)$ is approximately obtained by

*W*(*h*) =$\frac{\frac{d}{dt}k_{t}(h,i,j)}{V[k_{t}(h,i,j)]}$. (5)

(refer to APPENDIX).

In this study, we assume $\frac{d}{dt}k_{t}(h,i,j)$is proportional to time (molecular clock) for all pair of OTUs *i* and *j*. Namely,

$\frac{d}{dt}k_{t}(h,i,j)$= $u(h)$=$\frac{k_{t}(h,i,j)}{2t(i,j)}$, (6)

where $t(i,j)$is the divergence time between OTUs *i* and *j* and $u(h)$is the substitution rate of gene *h*. By using this relationship, $u(h)$can be estimated by

$u(h)=\frac{\sum_{i=1}^{n-1} \sum_{j=i+1}^{n} k_{t}(h,i,j)}{2\sum_{i=1}^{n-1} \sum_{j=i+1}^{n} t(i,j)}$, (7)

In most cases, however, the divergence time between OTUs *i* and *j* is not known, therefore, we obtained $W(h)$ by

$W(h)=\frac{C\sum_{i=1}^{n-1} \sum_{j=i+1}^{n} k_{t}(h,i,j)}{\sum_{i=1}^{n-1} \sum_{j=i+1}^{n} V[k_{t}(h,i,j)]}$, (8)

where *C* is given by

$C=\frac{1}{2\sum_{i=1}^{n-1} \sum_{j=i+1}^{n} t(i,j)}$, (9)

Since we assume the phylogenetic tree for all genes are the same, *C* is a constant for all genes, although gene trees sometimes differ from species tree (Maddison 1997). It is worth noting that *C* does not affect the reconstructing trees, because all distances are multiplied by *C.* Thus, in the calculation of$W(h)$, we do not have to calculate *C*. It is also worth noting that $W(h)$ is also an increasing function of the gene length, $l(h)$, because $V[k_{t}(h,i,j)]$ is inversely proportional to $l(h)$.

## Modified Least Square Method

We also developed another new method, namely, the modified least square method. This method is similar to the least square method, but it involves the use of a single weight for each locus based on the variances. By assigning 1/$\sum_{i=1}^{n-1} \sum_{j=i+1}^{n} V[k_{t}(h,i,j)]$ as the weight for locus *h*, the pooled distance between the OTUs *i* and *j*, i.e., $O(i,j)$, is obtained by the following equation:

$O(i,j)$ = $\sum_{h=1}^{N} \frac{k_{t}(h,i,j)}{\sum_{i=1}^{n-1} \sum_{j=i+1}^{n} V[k_{t}(h,i,j)]}$. (10)

## **Computer Simulations**

Computer simulations were conducted to compare the efficiencies of these weighting methods for phylogeny reconstruction. Since the efficiencies of the weighting methods would depend on the tree topology and branch lengths (Goldstein and Pollock 1994; Tajima and Takezaki 1994; Pollock and Goldstein 1995), the simulations were performed under various conditions.

## Simulating the Sequence Evolution

We used 2 model trees as shown in Fig. 1. T is the time unit in the simulation. Tree A is an asymmetric tree and tree B is a symmetric tree. These trees are basically the same as those used by Tateno, Nei, and Tajima (1982).

Let us define $u(h)$ as the substitution rate per T per site of gene *h*. The $u(h)$ value was assumed to be the same for all sites of gene *h*. In order to introduce heterogeneity in the evolutionary rate among genes, $u(h)$ was assumed to follow the gamma distribution (Yang 1996).

*f*[*u*(*h*)] = $\frac{b^{a}}{\Gamma(a)}\exp[-bu(h)]u(h)^{a-1}$, (11)

where *a* and *b* are the parameters that determine the shape of the gamma distribution. Let us denote the expected value of $u(h)$ as *u*. The expectation and variance of $u(h)$ are given by

$E[u(h)]$= *u* = $\frac{a}{b}$, and $V[u(h)]$ = $\frac{u^{2}}{a}$. (12)

Note that when the value of *a* is infinity, there is no rate variation among the loci. Gamma-distributed random numbers were generated by using the algorithm described by Ahrens and Dieter (1974). In the computer simulation, sequences of 10 loci were generated. We conducted 2 sets of computer simulations. One is to examine the effects of the rate variation and the weighting methods on phylogenetic tree reconstruction, and the other is to examine the effects of the average rate and the weighting methods on phylogenetic tree reconstruction. When the former was investigated by computer simulation, *u* was fixed to 0.5, and *a* was incremented by 0.1 from 0.1 to 1.9. When the latter was investigated by computer simulation, *u* was incremented by 0.1 from 0.1 to 1.9, and *a* was fixed to 0.5.

### Substitutions on Protein Sequences

We simulated the evolution of amino acid sequences using the model trees. The sequence length of each locus was 100 amino acids. First, the common ancestral sequence in the entire sample is determined by choosing 1 of 20 amino acids that have equal probability in each site. In other words, substitutions were generated by using the Jukes and Cantor (1969) model with 20 character states.

$p(u,t)=1-\frac{19}{20}\exp\left[-\frac{20}{19}ut\right]$, (13)

where $p(u,t)$ is the probability that one site has a different amino acid after the time *t* at a mutation rate of *u*.

### Substitutions on DNA Sequences

We also simulated DNA sequences using the model trees. The sequence length of each locus was 100 nucleotides, and the substitutions were determined using the Kimura 2-parameter model (Kimura 1980).

$P(u,t)=\frac{1}{4}\left\{1-2\exp\left[-2(\alpha+\beta)t\right]+\exp\left[-4\beta t\right]\right\}$ (14)

and $Q(u,t)=\frac{1}{2}\left\{1-\exp\left[-4\beta t\right]\right\}$ (15)

where $P(u,t)$is the probability that 1 site has a different nucleotide, and the difference is of the transition type after the time *t* at a substitution rate of *u*. $Q(u,t)$is the probability that 1 site has a different nucleotide, and the difference is of the transversion type after the time *t* at a mutation rate of *u*. Since the value of *u* equals $\alpha+2\beta$, we obtain $\alpha$ and $\beta$ by using the transition to transversion ratio *r*:

$\alpha=\frac{ur}{2+r}$ (16)

and $\beta=\frac{u}{2+r}$. (17)

In this study, *r* = 10 was assumed. This value is similar to the transition to transversion ratio in hominoid mitochondrial genes (Tamura and Nei 1993).

## Estimating $k(h,i,j)$and $V[k(h,i,j)]$

In this paper, we have used only 2 distances—the Poisson distance for protein sequences and the Kimura 2-pameter distance for DNA sequences.

### Poisson Distance

The number of amino acid substitutions per site between the 2 amino acid sequences can be estimated by

$k_{t}(h,i,j)$= -ln[1-$p(h,i,j)$], (18)

where $p(h,i,j)$ is the observed proportion of different sites between the OTUs *i* and *j* in the sequence of the gene *h* (Zuckerkandl and Pauling 1965). By using the Taylor series expansion, equation (18) can be expressed as

$k_{t}(h,i,j)$= $\sum_{m=1}^{\infty} \frac{p(h,i,j)^{m}}{m}$. (19)

Let us denote the observed number of differences between *i* and *j* on gene *h* as $q(h,i,j)$, such that $q(h,i,j)$is equal to $l(h)$ $p(h,i,j)$. Because $q(h,i,j)$ follows a binomial distribution with the parameters $l(h)$ and $p(h,i,j)$, the unbiased estimate of $p(h,i,j)^{m}$is $q(h,i,j)^{(m)}/l(h)^{(m)}$ when *m* < $q(h,i,j)$, where *x*(*^m^*) is defined as

$x^{(m)}$= $\frac{x!}{(x-m)!}$, (20)

which can be obtained by the Taylor-series expansion (Tajima 1993). Therefore, by not considering terms higher than the $q(h,i,j)$-th order, we obtain the following equation:

$k_{t}(h,i,j)$ = $\frac{q(h,i,j)^{(m)}}{ml(h)^{(m)}}$, (21)

which gives an almost unbiased estimate of the number of amino acid substitutions per site (Tajima 1993). The observed number of differences between *i* and *j* on gene *h* is $q(h,i,j)$such that $q(h,i,j)$equals to $l(h)$ $p(h,i,j)$. In this paper, we used equation (21) to estimate the Poisson distance between protein sequences instead of (18) because equation (21) gives the unbiased estimate of the number of amino acid substitutions per site.

### Sampling Variance of Poisson Distance

As described above, we assume that the number of substitutions at each site follows the Poisson distribution. The variance of the Poisson distance is obtained by

$V[k_{t}(h,i,j)]$ = $\frac{p(h,i,j)}{l(h)\{1-p(h,i,j)\}}$ (22)

(Kimura 1969). Then, $w[k(h,i,j)]$ can be obtained by

$w(h,i,j)$=$l(h)\left\{\frac{1}{p(h,i,j)}-1\right\}$ (23)

When 2 sequences are identical, equation (22) is not applicable because $p(h,i,j)$ is 0. It is also known that when the value of $p(h,i,j)$ is very small, the inverse of the average of $p(h,i,j)$ is a biased estimate of $\frac{1}{p(h,i,j)}$. Therefore, in this study we used Haldane’s (1956) correction to obtain $w(h,i,j)$.

$w[k_{t}(h,i,j)]$ = $l(h)\left\{\frac{l(h)}{l(h)p(h,i,j)+1}-1\right\}$ (24)

### Distance Determined by the Kimura 2-Parameter Method

Since transitions occur more frequently than transversions in DNA sequences, Kimura (1980) defined an evolutionary distance which can be given by

$k_{t}(h,i,j)$**=** $-\frac{1}{2}\ln[1-2P(h,i,j)-Q(h,i,j)]-\frac{1}{4}\ln[1-2Q(h,i,j)]$, (25)

where $P(h,i,j)$ and $Q(h,i,j)$ are the observed proportions of the transitional and transversional differences between the OTUs *i* and *j* on gene *h*, respectively (Kimura 1980).

When the observed numbers of transition-type and transversion-type pairs between 2 nucleotide sequences are denoted by $s(h,i,j)$*,* and $t(h,i,j)$*,* respectively, the evolutionary distance can be estimated by

$k_{t}(h,i,j)$**=** $\sum_{n=1}^{s(h,i,j)+t(h,i,j)} \frac{1}{nl(h)^{(n)}}\sum_{m=\min}^{\max} \left(\begin{matrix} n \\ m \end{matrix}\right)2^{m-1}s(h,i,j)^{(m)}t(h,i,j)^{(n-m)}$

$+\sum_{n=1}^{t(h,i,j)} \frac{2^{n-2}t(h,i,j)^{(n)}}{nl(h)^{(n)}}$, (26)

where min is the larger one of 0 and $l(h)-\{s(h,i,j)+t(h,i,j)\}$, max is the smaller one of *n* and $s(h,i,j)+t(h,i,j)$*,* and $\left(\begin{matrix} n \\ m \end{matrix}\right)=\frac{n!}{m!(n-m)!}$ (Tajima 1993). In this paper, we used (26) to estimate the Kimura 2-parameter distance between DNA sequences instead of (25) because (26) gives the unbiased estimate of the number of nucleotide substitutions per site

### Sampling Variance of the Distance Determined by the Kimura 2-Parameter Method

The variation of $k_{t}(h,i,j)$ is obtained by

$V[k_{t}(h,i,j)]$**=**$\frac{a^{2}P(h,i,j)+b^{2}Q(h,i,j)-\{aP(h,i,j)+bQ(h,i,j)\}^{2}}{l(h)}$, (27)

where $a=\frac{1}{1-2P(h,i,j)-Q(h,i,j)}$ and $b=\frac{1}{1-Q(h,i,j)}$ (Kimura 1980). In the case of DNA sequences, $w(h,i,j)$is obtained by $1/V[k(h,i,j)]$, without using Haldane’s (1956) correction because of the complexity of the equation (26).

## Pooling Distances

All the distances obtained by using the methods described above were pooled. We used the following 4 pooling methods: the no-weight method, least square method, modified Tajima-Takezaki method, and modified least square method.

## Tree Reconstruction

Finally, these pooled distances were used to reconstruct the phylogenetic trees by the neighbor-joining (NJ) method (Saitou and Nei 1987). The trial simulation was repeated 10,000 times for each set of parameters, and the proportion of trials that yielded the correct tree topology (PC) was obtained. We also compared the topological distances (Rzhetsky and Nei 1992) between the correct tree and the reconstructed tree (dT). For unrooted bifurcating trees, this distance is twice the number of different ways of partitioning sequences between 2 different trees. The smaller the topological distance between 2 trees, the greater is the similarity between the trees.

# Application in Hominoid Mitochondrial Phylogeny

Since the mitochondrial phylogeny in hominoid was well established (Horai et al. 1995), we reconstruct hominoid phylogeny by using mitochondrial genes to compare the methods described above. We used protein sequences as well as tRNA sequences of mitochondrial DNA (mtDNA) of four hominoid species, namely, orangutan (*Pongo pygmaeus abelii*), gorilla (*Gorilla gorilla*), bonobo (*Pan paniscus*), and human (*Homo sapiens*). The accession numbers for the mitochondrial DNA sequences of orangutan, gorilla, bonobo, and human are X97707, D38114, D38116, and D38112, respectively. Mitochondrial sequences were taken from the study by Takezaki and Gojobori (1999). Mitochondrial sequences were aligned by using the MAFFT program (Katoh et al. 2002). The complete deletion option (Nei and Kumar 2000) was used for the gapped sites in the reconstructed phylogenetic trees. Alignments are available at http://sourceforge.jp/projects/parallelgwas/releases/?package_id=9706. It should be noted that the lengths of 16S rRNA and 12S rRNA are about 1400bp and 900bp, respectively, while the lengths of tRNAs are less than 100bp, so that adding 16S rRNA and 12S rRNA would have larger effect on phylogenetic reconstruction than that of weighting methods. Thus, we excluded 16S rRNA and 12S rRNA when the weighting methods were compared.

To estimate the number of substitutions per site, equation (21) was used for protein sequences, and equation (26) was used for DNA sequences. To determine the pooled distances, four sets of methods, namely, the no-weight method, least square method, modified Tajima-Takezaki method, and modified least square method, were used. Gene names and gene lengths are shown in Table 2. The NJ trees were reconstructed using the pooled distances. We performed the bootstrap test for phylogenetic relationships (Felsenstein 1985). Bootstrap resampling was performed 10,000 times.

**References**

Ahrens JH, Dieter U (1974) Computer methods for sampling from gamma, beta, Poisson and bionomial distributions Computing 12:223-246

Felsenstein J (1985) Confidence limits on phylogenies: an approach using the bootstrap. Evolution 39:783-791

Goldstein DB, Pollock DD (1994) Least squares estimation of molecular distance--noise abatement in phylogenetic reconstruction. Theor Popul Biol 45:219-26

Haldane JBS (1956) The estimation of viabilities. J. Genet. 54:294-296

Horai S, Hayasaka K, Kondo R, Tsugane K, Takahata N (1995) Recent African origin of modern humans revealed by complete sequences of hominoid mitochondrial DNAs. Proc Natl Acad Sci U S A 92:532-6

Jukes TH, Cantor TH (1969) Evolution of protein molecules. In: Munro HN (ed) Mammalian Protein Metabolism. Academic Press, New York

Katoh K, Misawa K, Kuma K, Miyata T (2002) MAFFT: a novel method for rapid multiple sequence alignment based on fast Fourier transform. Nucleic Acids Res 30:3059-66

Kimura M (1969) The rate of molecular evolution considered from the standpoint of population genetics. Proc Natl Acad Sci U S A 63:1181-8

Kimura M (1980) A simple method for estimating evolutionary rates of base substitutions through comparative studies of nucleotide sequences. J Mol Evol 16:111-20

Lynch M (1999) The age and relationships of the major animal phyla. Evolution 53:319-325

Maddison WP (1997) Gene trees in species trees. Systematic Biology 46:523-536

Rzhetsky A, Nei M (1992) A simple method for estimating and testing minimum-evolution trees. Mol Biol Evol 9:945-967

Saitou N, Nei M (1987) The neighbor-joining method: a new method for reconstructing phylogenetic trees. Mol Biol Evol 4:406-25

Tajima F (1993) Unbiased estimation of evolutionary distance between nucleotide sequences. Mol Biol Evol 10:677-88

Tajima F, Takezaki N (1994) Estimation of evolutionary distance for reconstructing molecular phylogenetic trees. Mol Biol Evol 11:278-86

Takezaki N, Gojobori T (1999) Correct and incorrect vertebrate phylogenies obtained by the entire mitochondrial DNA sequences. Mol Biol Evol 16:590-601

Tamura K, Nei M (1993) Estimation of the number of nucleotide substitutions in the control region of mitochondrial DNA in humans and chimpanzees. Mol Biol Evol 10:512-26

Tateno Y, Nei M, Tajima F (1982) Accuracy of estimated phylogenetic trees from molecular data. I. Distantly related species. J Mol Evol 18:387-404

Yang Z (1996) Maximum-Likelihood Models for Combined Analyses of Multiple Sequence Data. J Mol Evol 42:587-96

Zuckerkandl E, Pauling L (1965) Evolutionary divergence and convergence in proteins. In: Bryson V, Vogel HJ (eds) Evolving genes and proteins. Academic Press, New York, p 97-166

APPENDIX: PROOF OF EQUATION (5)

Let $M(i,j)$ be the distance between OTUs *i* and *j* that is obtained by pooling (*h* = 1, …, *N*). We assume that each locus evolves independently so that there are no covariances among genes. Let us define $m(H,i,j)$by

$m_{t}(H,i,j)$ = $\sum_{h=1}^{H} W(h)k_{t}(h,i,j)$.

In other words, $m_{t}(H,i,j)$is the distance that has the largest accuracy index $A[m(H,i,j)]$among the pooled distances when genes 1, 2, …, *H* are used.

(*i*) When *N* = *H* = 1, $m(H,i,j)$ = $M(i,j)$ = $W(1)k_{t}(1,i,j)$. From equations (4) and (5), $A[m(1,i,j)]$ is calculated as

$A[m(1,i,j)]$ =

= = $A[k_{t}(1,i,j)]$. (A1)

Thus, equation (5) is true when *N* = 1. It is worth noting that the weight W(1) can be any number and any function, because W(1) appears in the numerator as well as in the denominator of (A1).

(*ii*) Let us assume that (5) is true for all *h* where 1 ≤ *h* ≤ *H*-1 < *N*. Consider a case where a new distance $k_{t}(H,i,j)$ is weighted by $W(H)$ and is added to $m_{t}(H-1,i,j)$.

$m_{t}(H,i,j)$ = $m_{t}(H-1,i,j)$*+*$W(H)k_{t}(H,i,j)$ (A2)

We need to find a value for $W(H)$ that yields the maximum value of $A[m_{t}(H,i,j)]$. From equation (5), we have

$A[m_{t}(H,i,j)]$ =$A[m_{t}(H-1,i,j)+W(H)k_{t}(H,i,j)]$

= $\frac{\frac{d}{dt}\left\{m_{t}(H-1,i,j)+W(H)k_{t}(H,i,j)\right\}}{\sqrt{V\left[m_{t}(H-1,i,j)+W(H)k_{t}(H,i,j)\right]}}$. (A3)

Since evolutionary distances must be an increasing function of divergence time and the variances of evolutionary distances are positive, the accuracy index$A[m_{t}(H,i,j)]$ defined by (3) is always positive. Thus, finding a $W(H)$that maximizes (A3) is equivalent to finding a $W(H)$that maximizes (A3) squared.

$\left\{A[m_{t}(H,i,j)]\right\}^{2}=\frac{\left[\frac{d}{dt}\left\{m_{t}(H-1,i,j)+W(H)k_{t}(H,i,j)\right\}\right]^{2}}{V\left[m_{t}(H-1,i,j)+W(H)k_{t}(H,i,j)\right]}$. (A4)

Since we assume that (5) is true for all *h* where 1 ≤ *h* ≤ *H*-1 < *N*, the numerator of (A4) is obtained by

$$\left[\frac{d}{dt}\left\{m_{t}(H-1,i,j)+W(H)k_{t}(H,i,j)\right\}\right]^{2}$$

$$=\left[\sum_{h}^{H-1} W(h)\frac{d}{dt}k_{t}(h,i,j)+W(H)\frac{d}{dt}k_{t}(H,i,j)\right]^{2}$$

$=\left[\sum_{h}^{H-1} \frac{\left\{\frac{d}{dt}k_{t}(h,i,j)\right\}^{2}}{V[k_{t}(h,i,j)]}+W(H)\frac{d}{dt}k_{t}(H,i,j)\right]^{2}$ . (A5)

Since we assume that (5) is true for all cases where 1 ≤ *h* ≤ *H*-1 < *N* and that the distance among genes have no covariances, the denominator of (A3) is obtained by

$$V\left[m_{t}(H-1,i,j)+W(H)k_{t}(H,i,j)\right]$$

$=\sum_{h}^{H-1} \left\{W(h)\right\}^{2}V[k_{t}(h,i,j)]+\left\{W(H)\right\}^{2}V[k_{t}(H,i,j)]$ $=\sum_{h}^{H-1} \frac{\left\{\frac{d}{dt}k_{t}(h,i,j)\right\}^{2}}{V[k_{t}(h,i,j)]}+\left\{W(H)\right\}^{2}V[k_{t}(H,i,j)]$ . (A6)

Let us define $c$ by

$c=\sum_{h}^{H-1} \frac{\left\{\frac{d}{dt}k_{t}(h,i,j)\right\}^{2}}{V[k_{t}(h,i,j)]}$. (A7)

Then, we obtain

$\left\{A[m_{t}(H,i,j)]\right\}^{2}=\frac{\left\{c+W(H)\frac{d}{dt}k_{t}(H,i,j)\right\}^{2}}{c+W(H)^{2}V[k_{t}(H,i,j)]}$. (A8)

$W(H)$can be obtained by solving the following differentiate equation:

$\frac{\partial}{\partial W(H)}\left\{A[m_{t}(H,i,j)]\right\}^{2}=\frac{\partial}{\partial W(H)}\frac{\left\{c+W(H)\frac{d}{dt}k_{t}(H,i,j)\right\}^{2}}{c+W(H)^{2}V[k_{t}(H,i,j)]}=0$. (A9)

There are two solutions for equation (32) as follows:

$W(H)=-\frac{d}{dt}k_{t}(H,i,j)/c,\frac{\frac{d}{dt}k_{t}(H,i,j)}{V[k_{t}(H,i,j)]}$ (A10)

The second derivative of (A8) shows that (A8) has the maximum value when

$W(H)=\frac{\frac{d}{dt}k_{t}(H,i,j)}{V[k_{t}(H,i,j)]}$ . (A11)

Thus, equation (5) holds true for *H* when we assume that (5) is true for all cases where 1 ≤ *H*-1 <*N*. On the basis of mathematical induction, (*i*) and (*ii*) yields

$M(i,j)$= $\sum_{h=1}^{N} W(h)k_{t}(h,i,j)$. (A12)

| Supplementary Table 1. List of Variables | | |  |  |
| --- | --- | --- | --- | --- |
| Variables |  | Explanation |  | Equations |
| α and β |  | Parameters of Kimura's (1980) 2-parameter Model |  | (14) to (15) |
| *a* and *b* |  | The Parameter of the Gamma Distribution |  | (11) and (12) |
| *A*[*k_t_*(*h*, *i*, *j*)] |  | The Accuracy Index of *k_t_*(*h*, *i*, *j*) |  | (3) |
| *C* |  | The Sum of Divergence Times of All Pairs of OTUs. |  | (8) and (9) |
| *h* |  | An Identifier for a Gene |  | (1) |
| *i* |  | An Identifier for an OTU |  | (1) |
| *j* |  | Another Identifier for an OTU |  | (1) |
| *k_t_*(*h*, *i*, *j*) |  | The Estimated Number of Substitutions per Site between OTU *i* and OTU *j* on Gene *h*. |  | (1) |
| *K*(*i*, *j*) |  | The Total Number of Substitutions between OTUs i and j. |  | (1) |
| *l*(*h*) |  | The Length of Gene *h*. |  | (1) |
| *L*(*i*, *j*) |  | The Distance between OTUs *i* and *j* Obtained by the Least Square Method |  | (2) |
| *M*(*i*, *j*) |  | The Distance between OTUs *i* and *j* that Appoximately Maximizes *A*[*k_t_*(*h*, *i*, *j*)] |  | (4) |
| *N* |  | Number of Genes |  | (1) |
| *n* |  | Number of OTUs |  | (1) |
| *O*(*i*, *j*) |  | The Distance between OTUs *i* and *j* Obtained by the Modified Least Square Method |  | (10) |
| *p*(*u, t*) |  | The Probability that One Site Has a Different Amino Acid after the Time *t* when the Mutation Rate Is *u* |  | (13) |
| *P*(*u, t*) |  | The Probability that One Site Has a Different Nucleotide and the Difference is the Transition-type after the Time *t* when the Mutation Rate Is *u* |  | (14) |
| *p*(*h*, *i*, *j*) |  | The Observed Proportion of Different Sites between OTUs *i* and *j* on Gene *h*. |  | (18) |
| *P*(*h*, *i*, *j*) |  | The Observed Proportion of Transition-type Pairs between OTUs *i* and *j* on Gene *h*. |  | (25) |
| *Q*(*u, t*) |  | The Probability that One Site Has a Different Nucleotide and the Difference is the Transition-typeafter the Time *t* when the Mutation Rate Is *u* |  | (15) |
| *Q*(*h*, *i*, *j*) |  | The Observed Proportion of Transversion-type Pairs between OTUs *i* and *j* on Gene *h*. |  | (25) |
| *r* |  | The Ratio of Transitions to Transversions |  | (16) and (17) |
| *s*(*h*, *i*, *j*) |  | The Observed Number of Transition-type Difference between OTUs *i* and *j* on Gene *h*. |  | (26) |
| *t*(*h*, *i*, *j*) |  | The Observed Number of Transversion-type Difference between OTUs *i* and *j* on Gene *h*. |  | (26) |
| *T* |  | Time Unit for Model Trees |  | Figure 1 |
| *u* |  | The Average Mutation Rate |  | (12) |
| *u*(*h*) |  | Mutation Rate per Site per *T* of Gene *h* |  | (7) and (11) |
| *V*[*k_t_*(*h*, *i*, *j*)] |  | The Estimated Variance of *k*(*h*, *i*, *j*) |  | (2), (22) and (27) |
| *w*(*h*) |  | The Weight for OTUs *i* and *j* of Gene *h* for the Least Square Method |  | (2) |
| *W*(*h*) |  | The Weight for Gene *h* that Appoximately Maximizes *A*[*k_t_*(*h*, *i*, *j*)] |  | (8) |
| *x* |  | Another Identifier for an OTU |  | (1) |
| *y* |  | Another Identifier for an OTU |  | (1) |

| Supplementary Table 2. Weights for Mitochondrial Genes Obtained by New Methods | | | | |
| --- | --- | --- | --- | --- |
| Gene Name | Estimated Number of Substitutions per Site between Orangutan and Human | Gene length | Weight Obtained by the Modified Tajima-Takezaki Method | Weight Obtained by the Modified Least Square Method |
| Proteins | | | | |
| atp6 | 0.22 | 217 | 27.28 | 29.17 |
| atp8 | 0.43 | 50 | 3.75 | 2.05 |
| co1 | 0.04 | 509 | 81.56 | 526.87 |
| co2 | 0.05 | 225 | 35.91 | 207.29 |
| co3 | 0.08 | 258 | 39.89 | 135.00 |
| cytb | 0.13 | 376 | 54.62 | 92.80 |
| nd1 | 0.15 | 312 | 43.50 | 68.12 |
| nd2 | 0.16 | 339 | 47.12 | 73.20 |
| nd3 | 0.18 | 110 | 14.74 | 19.81 |
| nd4 | 0.12 | 453 | 66.33 | 132.54 |
| nd4l | 0.08 | 91 | 13.85 | 48.59 |
| nd5 | 0.18 | 583 | 77.51 | 91.40 |
| nd6 | 0.05 | 136 | 21.28 | 82.05 |
| tRNAs | | | | |
| ALA | 0.05 | 64 | 9.71 | 50.68 |
| ARG | 0.08 | 63 | 9.07 | 27.18 |
| ASN | 0.11 | 69 | 9.20 | 16.75 |
| ASP | 0.18 | 65 | 7.68 | 10.00 |
| CYS | 0.07 | 64 | 9.14 | 26.47 |
| GLN | 0.09 | 69 | 9.72 | 31.75 |
| GLU | 0.22 | 66 | 8.63 | 10.33 |
| GLY | 0.06 | 67 | 8.82 | 15.46 |
| HIS | 0.14 | 68 | 9.61 | 20.26 |
| ILE | 0.09 | 69 | 10.00 | 27.41 |
| LEU(CUN) | 0.03 | 60 | 9.35 | 46.02 |
| LEU(UUR) | 0.07 | 71 | 9.95 | 31.73 |
| LYS | 0.09 | 59 | 8.66 | 25.88 |
| MET | 0.00 | 68 | 20.31 | 148.94 |
| PHE | 0.16 | 63 | 8.94 | 15.92 |
| PRO | 0.19 | 64 | 8.50 | 13.72 |
| SER(AGY) | 0.08 | 53 | 6.58 | 7.83 |
| SER(UCN) | 0.08 | 66 | 9.46 | 27.06 |
| THR | 0.31 | 61 | 5.48 | 3.02 |
| TRP | 0.20 | 61 | 8.14 | 11.23 |
| TYR | 0.10 | 64 | 8.33 | 15.76 |
| VAL | 0.18 | 63 | 7.95 | 12.84 |
