# Supplementary material for: New Weighting Methods for Phylogenetic Tree Reconstruction Using Multiple Loci
Source: J Mol Evol. 2012 Aug 8;75(1):1–10. doi: 10.1007/s00239-012-9513-4 (PMC3480593; doi:10.1007/s00239-012-9513-4)

Supplementary Figure Legend

Supplementary Figure S1 Topological distance (dT) between the correct tree and the reconstructed tree obtained by using the no-weight method (filled triangle, ▲), the least square method (open square, □), the modified Tajima-Takezaki method (filled circle, ●), and the modified least square method (cross, ×) when protein sequences were simulated based on trees A and B and the Poisson distances are used. In this Supplementary Figure, *u* was fixed to 0.5 *a* was incremented, where *u* is the average mutation rate and *a* is the gamma-shape parameter as described in table 1. 99% confidence intervals are also shown.

Supplementary Figure S2. Topological distance (dT) between the correct tree and the reconstructed tree obtained by using the no-weight method (filled triangle, ▲), the least open square method (open square, □), the modified Tajima-Takezaki method (filled circle, ●) , and the modified least square method (cross, ×) when DNA sequences were simulated based on trees A and B and the Kimura's (1980) 2-parameter distances are used. In this Supplementary Figure, *u* was fixed to 0.5 *a* was incremented, where *u*

is the average mutation rate and *a* is the gamma-shape parameter as described in table 1. 99% confidence intervals are also shown.

Supplementary Figure S3. Topological distance (dT) between the correct tree and the reconstructed tree obtained by using the no-weight method (filled triangle, ▲), the least open square method (open square, □), t modified Tajima-Takezaki method (filled circle, ●), and the modified least square method (cross, ×) when protein sequences were simulated based on trees A and B and the Poisson distances are used. In this Supplementary Figure, *u* was incremented and *a* was fixed to 0.5, where *u* is the average mutation rate and *a* is the gamma-shape parameter as described in table 1. 99% confidence intervals are also shown.

Supplementary Figure S4. Topological distance (dT) between the correct tree and the reconstructed tree obtained by using the no-weight method (filled triangle, ▲), the least open square method (open square, □), modified Tajima-Takezaki method (filled circle, ●), and the modified least square method (cross, ×) when DNA sequences were

simulated based on trees A and B and the Kimura's (1980) 2-parameter distances are used. In this Supplementary Figure, *u* was incremented and *a* was fixed to 0.5, where *u* is the average mutation rate and *a* is the gamma-shape parameter as described in table 1. 99% confidence intervals are also shown.

Supplementary Figure S5. Proportion of trials that yielded the correct tree topology (PC) by using the no-weight method (filled triangle, ▲), the least square method (open square, □), the modified Tajima-Takezaki method (filled circle, ●), and the modified least square method (cross, ×) when protein sequences were simulated and the Poisson distances are used. In this figure, *u* was fixed to 0.5 *a* was incremented, where *u* is the average mutation rate and *a* is the gamma-shape parameter as described in table 1. Tree Au is topologically the same as Tree A, but its branch lengths are given by uniformly distributed random numbers. The expectations of the uniformly distributed random numbers are the same as the branch lengths of corresponding branches of Tree A. Tree Bu is topologically the same as Tree B, but its branch lengths are given by uniformly distributed random numbers. The expectations of the uniformly distributed random numbers are the same as the branch lengths of corresponding branches of Tree B. 99% confidence intervals are also shown.

Supplementary Figure S6. Proportion of trials that yielded the correct tree topology (PC) by using the no-weight method (filled triangle, ▲), the least open square method (open square, □), the modified Tajima-Takezaki method (filled circle, ●) , and the modified least square method (cross, ×) when DNA sequences were simulated based on trees Au and Bu and the Kimura's (1980) 2-parameter distances are used. In this figure, *u* was fixed to 0.5 *a* was incremented, where *u* is the average mutation rate and *a* is the gamma-shape parameter as described in table 1. 99% confidence intervals are also shown.

Supplementary Figure S7. Proportion of trials that yielded the correct tree topology (PC) by using the no-weight method (filled triangle, ▲), the least open square method (open square, □), t modified Tajima-Takezaki method (filled circle, ●), and the modified least square method (cross, ×) when protein sequences were simulated based on trees Au and Bu and the Poisson distances are used. In this figure, *u* was incremented and *a* was fixed to 0.5, where *u* is the average mutation rate and *a* is the

gamma-shape parameter as described in table 1.　99% confidence intervals are also shown.

Supplementary Figure S8. Proportion of trials that yielded the correct tree topology (PC) by using the no-weight method (filled triangle, ▲), the least open square method (open square, □), modified Tajima-Takezaki method (filled circle, ●), and the modified least square method (cross, ×) when DNA sequences were simulated based on trees Au and Bu and the Kimura’s (1980) 2-parameter distances are used.　In this figure, *u* was incremented and *a* was fixed to 0.5, where *u* is the average mutation rate and *a* is the gamma-shape parameter as described in table 1.　99% confidence intervals are also shown.

Supplementary Figure S9. Proportion of trials that yielded the correct tree topology (PC) by using the no-weight method (filled triangle, ▲), the least square method (open square, □), the modified Tajima-Takezaki method (filled circle, ●), and the modified least square method (cross, ×) when protein sequences were simulated based on trees

A and B and the Poisson distances are used. In this figure, *u* was fixed to 0.1 and *a* was incremented, where *u* is the average mutation rate and *a* is the gamma-shape parameter as described in table 1. 99% confidence intervals are also shown.

Supplementary Figure S10. Proportion of the cases in which the correct topology were reconstructed by using the no-weight method (filled triangle, ▲), the least open square method (open square, □), the modified Tajima-Takezaki method (filled circle, ●) , and the modified least square method (cross, ×) when DNA sequences were simulated based on trees A and B and the Kimura's (1980) 2-parameter distances are used. In this figure, *u* was fixed to 0.1 and *a* was incremented, where *u* is the average mutation rate and *a* is the gamma-shape parameter as described in table 1. 99% confidence intervals are also shown.

Supplementary Figure S11. Proportion of trials that yielded the correct tree topology (PC) by using the no-weight method (filled triangle, ▲), the least square method (open square, □), the modified Tajima-Takezaki method (filled circle, ●), and the modified

least square method (cross, ×) when protein sequences were simulated based on trees Au and Bu and the Poisson distances are used. In this figure, *u* was fixed to 0.1 and *a* was incremented, where *u* is the average mutation rate and *a* is the gamma-shape parameter as described in table 1. 99% confidence intervals are also shown.

Supplementary Figure S12. Proportion of trials that yielded the correct tree topology (PC) by using the no-weight method (filled triangle, ▲), the least open square method (open square, □), the modified Tajima-Takezaki method (filled circle, ●) , and the modified least square method (cross, ×) when DNA sequences were simulated based on trees Au and Bu and the Kimura’s (1980) 2-parameter distances are used. In this figure, *u* was fixed to 0.1 and *a* was incremented, where *u* is the average mutation rate and *a* is the gamma-shape parameter as described in table 1. 99% confidence intervals are also shown.

Supplemental Figure S1

Protein Sequences, Tree A

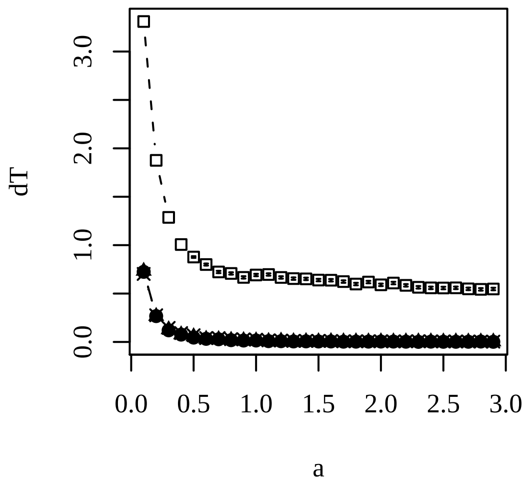

Protein Sequences, Tree B

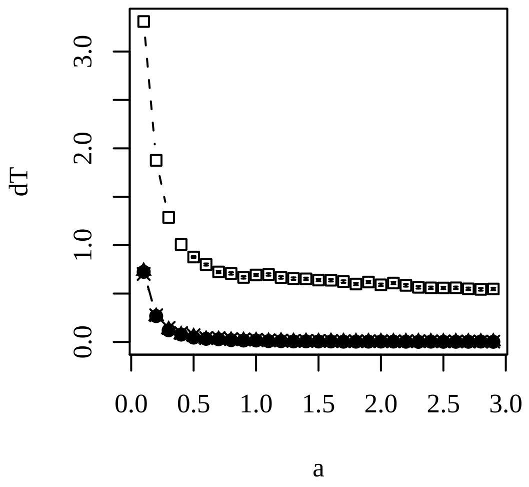

Supplemental Figure S2

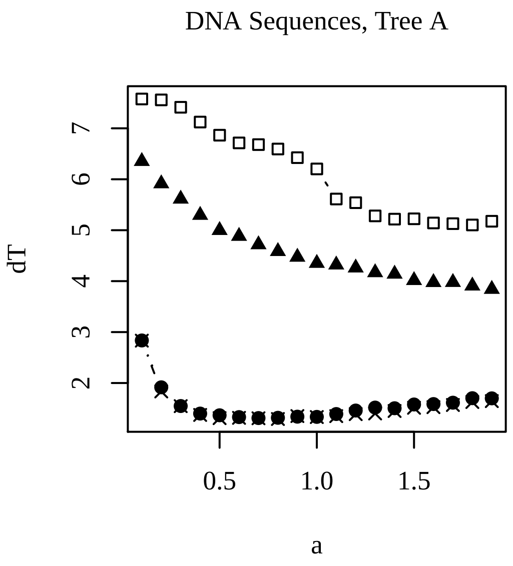

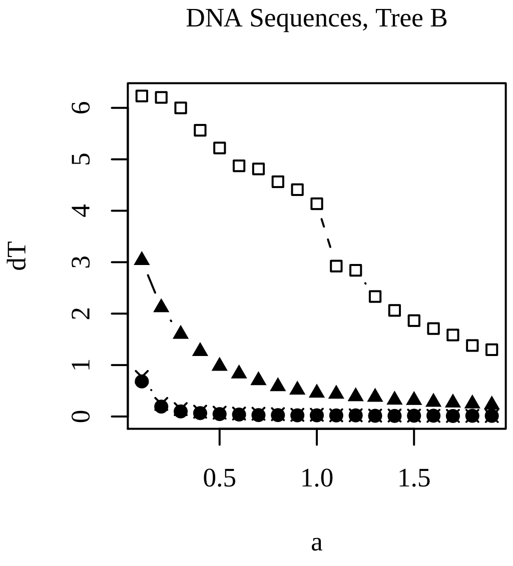

Supplemental Figure S3

Protein Sequences, Tree A

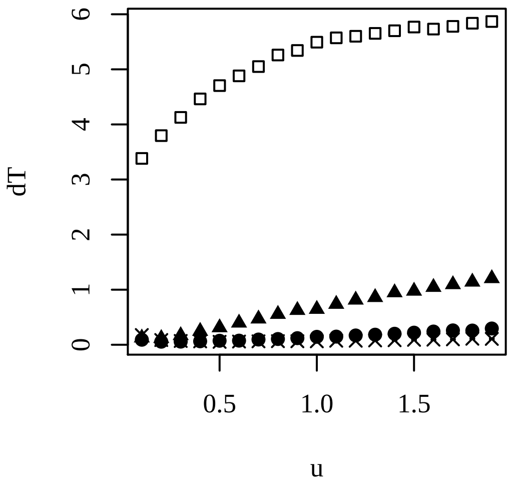

Protein Sequences, Tree B

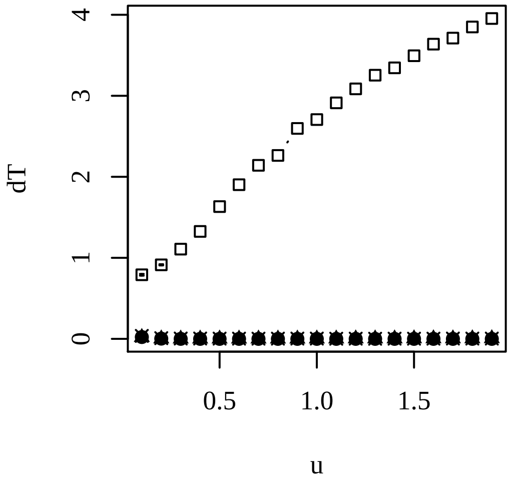

Supplemental Figure S4

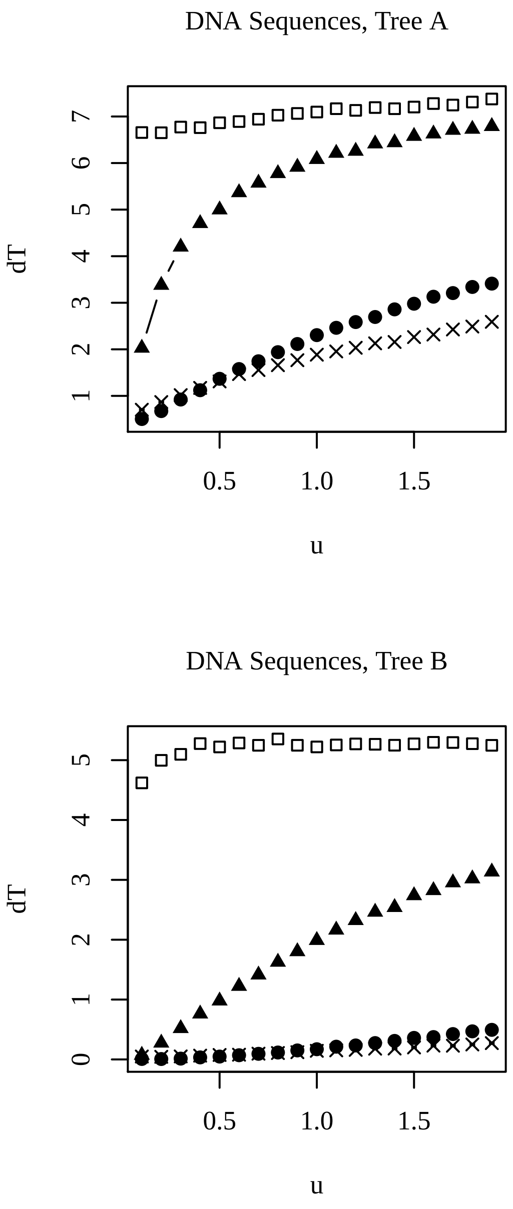

Supplementary Figure S5

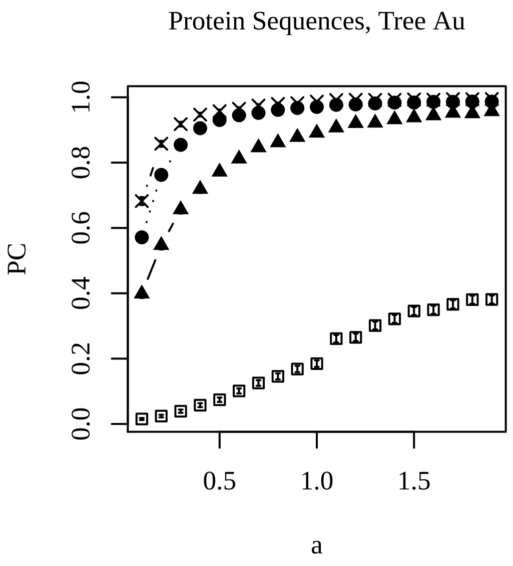

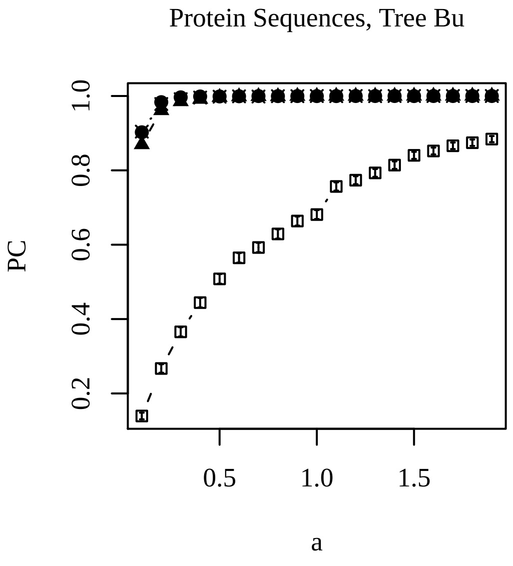

Supplementary Figure S6

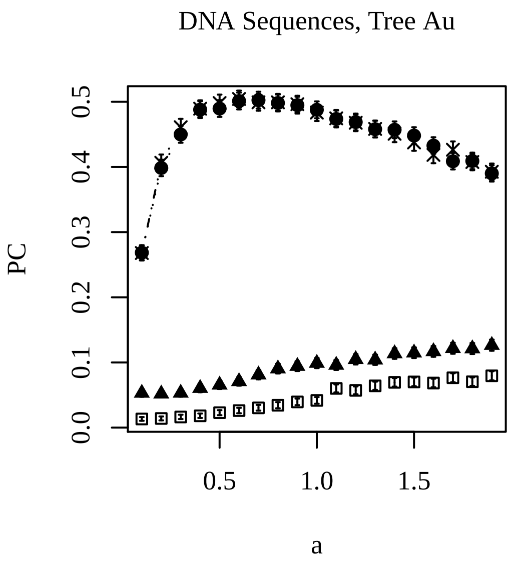

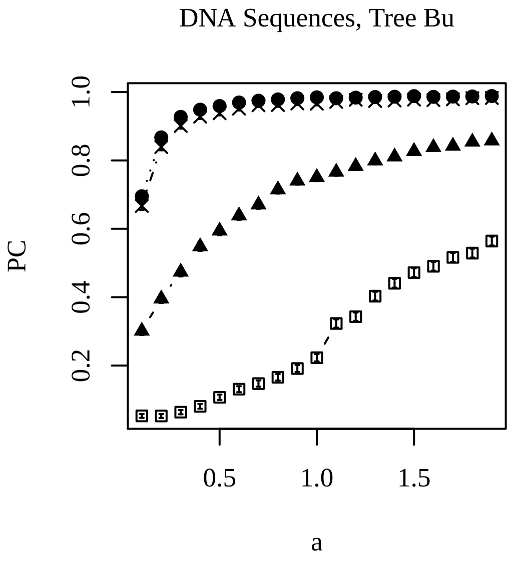

Supplementary Figure S7

Protein Sequences, Tree Au

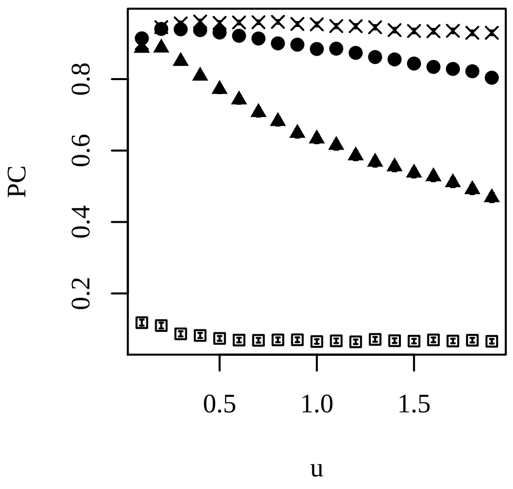

Protein Sequences, Tree Bu

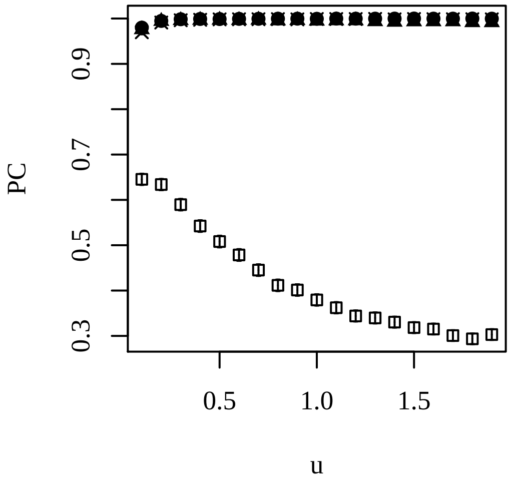

Supplementary Figure S8

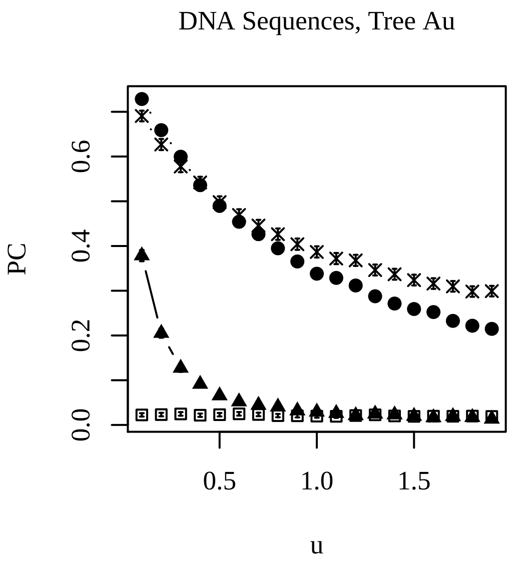

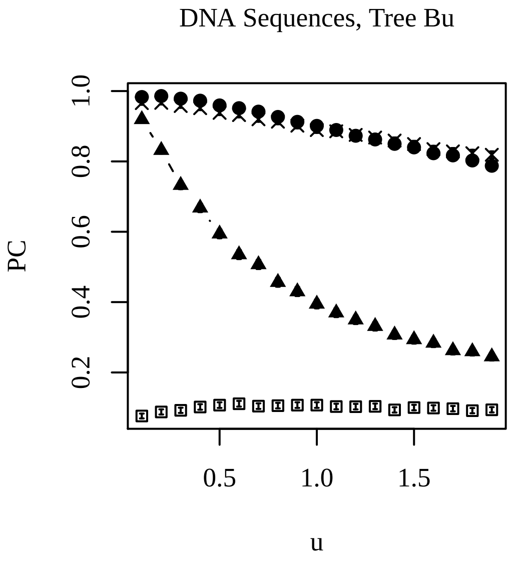

Supplementary Figure S9

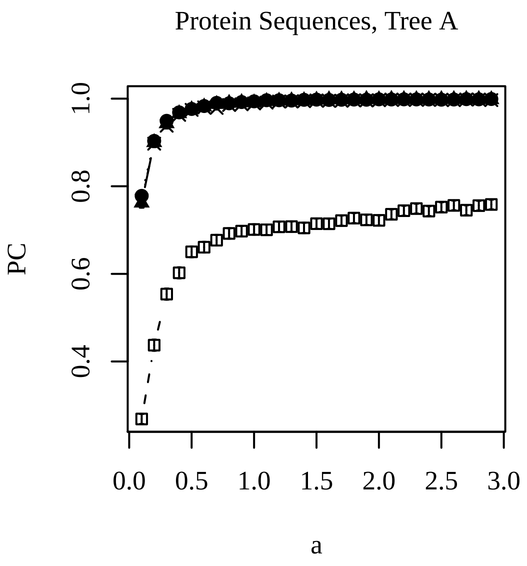

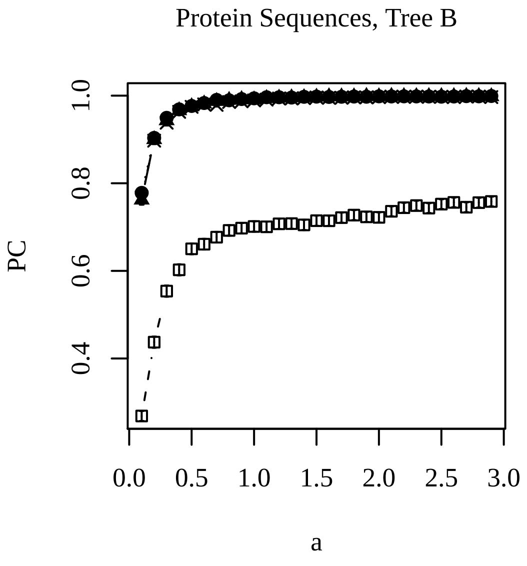

Supplementary Figure S10

DNA Sequences, Tree A

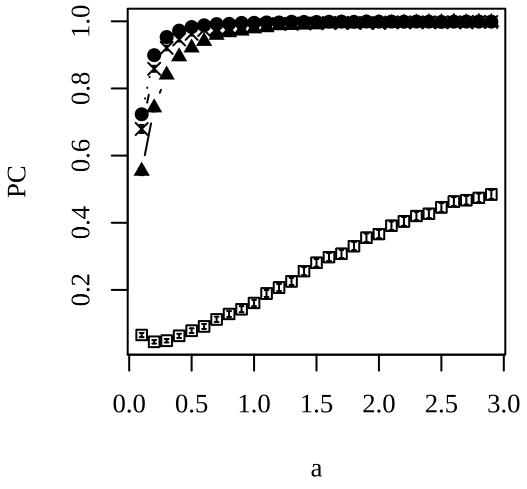

DNA Sequences, Tree B

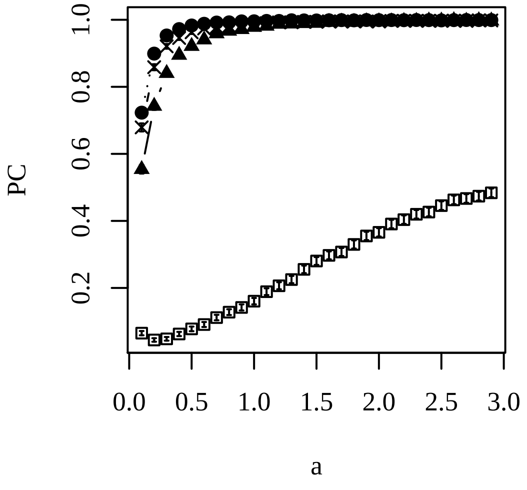

Supplementary Figure S11

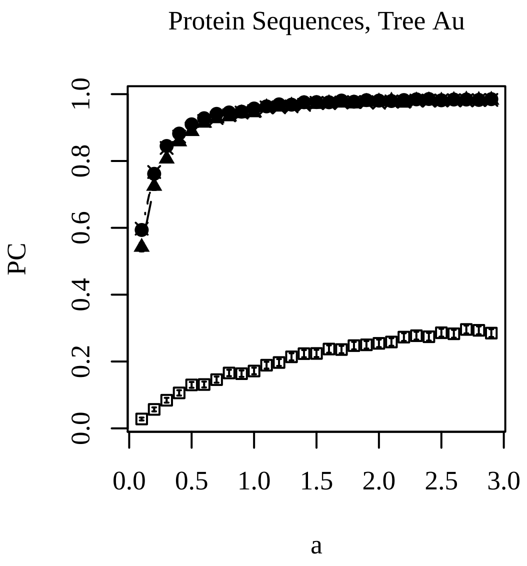

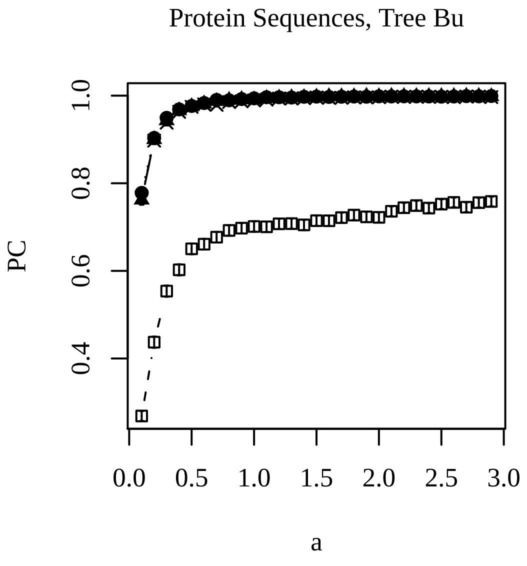

Supplementary Figure S12

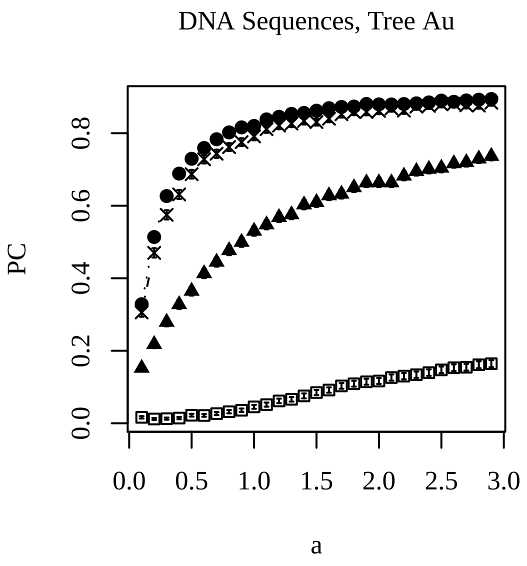

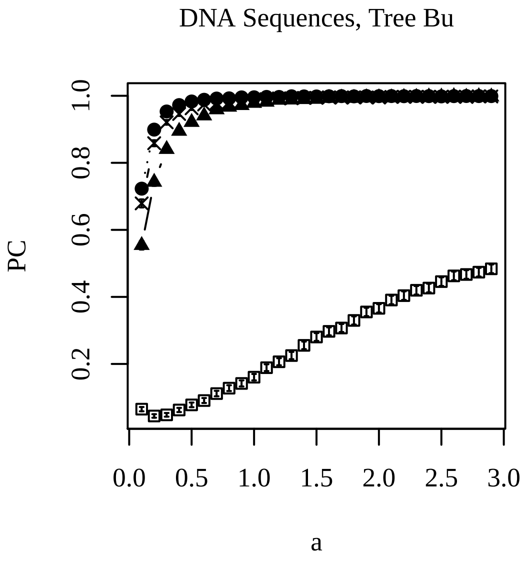

Supplement: Supplementary file 2 — Supplementary material 2 (PDF 189 kb) [file 239_2012_9513_MOESM2_ESM.pdf]
